# SOX2 interacts with hnRNPK to modulate alternative splicing in mouse embryonic stem cells

**DOI:** 10.1186/s13578-024-01284-8

**Published:** 2024-08-19

**Authors:** Yanlan Huang, Yuxuan Liu, Mingyi Pu, Yuli Zhang, Qiang Cao, Senru Li, Yuanjie Wei, Linlin Hou

**Affiliations:** 1 School of Medicine, Shenzhen Campus of Sun Yat-Sen University, Shenzhen, 518107 People’s Republic of China; 2 Helmholtz Centre for Infection Research (HZI), Helmholtz Institute for RNA-Based Infection Research (HIRI), Würzburg, Germany

## Abstract

**Background:**

SOX2 is a determinant transcription factor that governs the balance between stemness and differentiation by influencing transcription and splicing programs. The role of SOX2 is intricately shaped by its interactions with specific partners. In the interactome of SOX2 in mouse embryonic stem cells (mESCs), there is a cohort of heterogeneous nuclear ribonucleoproteins (hnRNPs) that contributes to multiple facets of gene expression regulation. However, the cross-talk between hnRNPs and SOX2 in gene expression regulation remains unclear.

**Results:**

Here we demonstrate the indispensable role of the co-existence of SOX2 and heterogeneous nuclear ribonucleoprotein K (hnRNPK) in the maintenance of pluripotency in mESCs. While hnRNPK directly interacts with the SOX2-HMG DNA-binding domain and induces the collapse of the transcriptional repressor 7SK small nuclear ribonucleoprotein (7SK snRNP), hnRNPK does not influence SOX2-mediated transcription, either by modulating the interaction between SOX2 and its target *cis*-regulatory elements or by facilitating transcription elongation as indicated by the RNA-seq analysis. Notably, hnRNPK enhances the interaction of SOX2 with target pre-mRNAs and collaborates with SOX2 in regulating the alternative splicing of a subset of pluripotency genes.

**Conclusions:**

These data reveal that SOX2 and hnRNPK have a direct protein-protein interaction, and shed light on the molecular mechanisms by which hnRNPK collaborates with SOX2 in alternative splicing in mESCs.

**Supplementary Information:**

The online version contains supplementary material available at 10.1186/s13578-024-01284-8.

## Background

Transcription factors serve as master regulators of cellular identity, with the ability to orchestrate cell fate transitions, such as differentiations, reprogramming, and transdifferentiations [1, 2]. Emerging evidence underscores their capacity to act as mediators in multiple facets of gene expression.

The High-mobility group (HMG)-box transcription factor SOX2 is one of the key transcription factors that play a crucial role in maintaining pluripotency of stem cells. Within the context of pluripotency, its HMG domain selectively binds to nucleosomal entry-exit sites [3]. In collaboration with OCT4, SOX2 induces a notable ~ 90° DNA distortion away from the histone octamer, initiating chromatin opening by leveraging binding energy [4]. This process enhances the accessibility of nucleosomal DNA and triggers nucleosome-mediated cooperativity among transcription factors or regulators, leading to robust activation of pluripotent gene expression while simultaneously repressing genes associated with differentiation, or vice versa [5–7].

Apart from its well-established roles in direct DNA-level transcriptional regulation, SOX2 has recently been discovered to participate in RNA-related processes that significantly impact cell fate determination [8, 9]. Despite lacking conventional RNA-binding motifs, SOX2 has been identified as an RNA-binding protein. It recognizes G/C-rich RNA sequences through a 60-amino-acid RNA binding motif (RBM) located C-terminally adjacent to the HMG domain (amino acids 120–180) [9, 10]. The HMG domain also exhibits RNA-binding activity, but in a non-sequence-specific manner [9, 11]. In the presence of both DNA and RNA, the RBM preferably binds RNA, while the HMG domain interacts with DNA predominately [9]. The RBM is suggested to regulate alternative splicing of pre-mRNA from genes that play essential roles in cell fate determination [9]. Deletion of the RBM leads to altered splicing site selection in multiple genes bound by SOX2, especially in exons rich in G/C sequences near the 5’ splice site [9]. This disruption ultimately impacts the efficiency of somatic cell reprogramming, indicating a role for SOX2 in alternative splicing regulation [9]. RNA-seq analysis has further confirmed that the RBM controls splicing patterns, independently of the HMG domain, for specific genes [9], underlining SOX2’s role in splicing regulation that is distinct from its HMG domain-related functions. This is consistent with the observation that more than one-third of alternative regulators are transcription factors, including those containing HMG domains [12]. SOX2 is believed to regulate the alternative splicing of pre-mRNA in genes pivotal for cell fate determination by associating with splicing factors within the SOX2 interactome [9, 12–15].

Heterogeneous nuclear ribonucleoproteins (hnRNPs) constitute a significant portion of the SOX2 interactome [13, 14, 16]. This group of RNA-binding proteins is known for recognizing specific RNA sequences and is commonly associated with various RNA metabolism processes, including pre-mRNA splicing, transcription, and translation regulation [17, 18]. Among these hnRNPs, hnRNPK actively contributes to pivotal physiological and pathological processes in pluripotent stem cells and development. hnRNPK binds to and modulates the alternative splicing of the *RUNX1* transcript, exerting a subsequent impact on myeloid development [19]. Additionally, hnRNPK recruits lncRNA-Smad7 to the Bmp2 promoter, suppressing Bmp2 expression, and hinders cardiomyocyte differentiation from mouse embryonic stem cells (mESCs) [20]. hnRNPK actively contributes to preventing premature differentiation of human epidermal progenitor cells by degrading mRNAs encoding differentiation-promoting transcription factors through the DDX6 pathway [21]. Our prior investigation revealed that hnRNPK is a part of the interactome with SOX2 and several splicing factors during reprogramming [9]. Bakhmet et al. discovered frequent co-occupation of hnRNPK by vital pluripotency-related transcription factors, including SOX2, alongside active histone marks within open chromatin regions in mESCs [22]. These observations suggest a potential coordination between hnRNPK and SOX2 in the regulation of pluripotency-related gene expression at the levels of splicing and/or transcription. However, the specifics of whether and how hnRNPK participates in cross-talk with SOX2 in gene expression regulation remain undocumented.

In this study, we elucidate a collaborative role of hnRNPK with SOX2 in maintaining pluripotency in mESCs. We observed the association of SOX2 with hnRNPK and their co-localization on chromatin in the pluripotent state. Despite the direct interaction of hnRNPK with the SOX2-HMG DNA-binding domain and its role in degrading the transcription repressor 7SK snRNPs, hnRNPK is dispensable for SOX2-mediated transcription. Instead, hnRNPK affects SOX2-mediated splicing of specific pluripotency-related gene transcripts. This effect is achieved by hnRNPK through enhancing the binding of SOX2 to its target pre-mRNAs. This study highlights a hitherto unknown interplay between SOX2 and hnRNPK in pre-mRNA splicing.

## Methods

### Cell culture

NIH3T3 and HEK293T cells were maintained in Dulbecco’s modified Eagle’s medium (DMEM) supplied with 10% FBS (Every Green, Cat#11011–8611) and 100 mg/ml penicillin–streptomycin (NCM Biotech, Cat#C100C5). E14Tg2A (E14) embryonic stem cells (ESCs) were cultured on 0.2% gelatin-coated plates in standard mouse embryonic stem cells medium (DMEM/high glucose (Hyclone, Cat#SH30022-2B) containing 15% FBS (Natocor, Cat#SFBE), 1 × GlutaMax (Gibco, Cat#35050079), 1 × MEM nonessential amino acids (Corning, Cat#25-025-CI), 0.055 mM 2-Mercaptoethanol (Gibco, Cat#21985023), 0.5 × penicillin/streptomycin (Hyclone, Cat#SV30010), with the presence of 1000 U/ml leukemia inhibitory factor (LIF, Merck, Cat#ESG1107), 3 mM CHIR99021 (Merck, Cat#252917), 1 mM PD0325901 (Merck, Cat#391210–10-9)). All cell lines were cultivated in a humidified incubator with 5% CO_2_ at 37 °C.

### Retinoic acid (RA)-induced differentiation of ESCs

E14 ESCs were seeded at a density of 0.3 × 10^6^ cells per 10 cm dish. After 24 h, LIF was withdrawn, and retinoic acid (RA, Merck, Cat#302-79-4) was introduced at a concentration of 1 µM. Cell samples were collected 24 h, 48 h and 72 h post-treatment.

### Chromatin-binding assay

The chromatin binding assay was conducted following established protocols with modifications [23, 24]. In brief, approximately 1 × 10^7^ E14 ESCs, with or without RA treatment, were lysed for 15 min on ice in cold CSK I buffer (10 mM Pipes, pH 6.8, 100 mM NaCl, 1 mM EDTA, 300 mM sucrose, 1 mM MgCl_2_, 1 mM DTT) supplemented with 0.5% (v/v) Triton X-100, protease inhibitors (MedChemExpress, Cat#HY-K0010) and 1 mM PMSF. The cell lysate was centrifuged at 500 g at 4 °C for 3 min. The pellet was resuspended in CSK II buffer (10 mM Pipes, pH 6.8, 50 mM NaCl, 300 mM sucrose, 6 mM MgCl_2_, 1 mM DTT), treated with DNase I (ThermoFisher Scientific, Cat#EN0521) in the presence of (NH_4_)_2_SO_4_ at 37 °C for 30 min to isolate the soluble chromatin fraction. After centrifugation at 1200 g for 6 min at 4 °C, the supernatant was collected and analyzed by Western blot using antibodies (anti-SOX2, Abcam, Cat#ab97959; anti-hnRNPK, ABclonal, Cat#A1701; anti-Histone H3, Cell Singaling Technology, Cat#96C10).

### Transfection and lentiviral infection

Cell transfection utilized Lipofectamine 3000 (Yeasen, Cat#40802ES03) following the manufacturer’s instructions. Protein expression was suppressed using the lentiviral vector GV248 (CON077) expressing specific shRNA. Lentiviral particles were generated by transfecting 293 T cells with 5 μg each of psPAX2, pMD2.G, and GV248, containing either the shRNA or a scramble control. The detailed sequences are listed in Additional file 1: Table S1. For lentivirus infection, E14 ESCs were trypsinized, re-suspended in standard mouse embryonic stem cells medium at a concentration of 0.25 × 10^6^ /ml. Cells (2 ml) were mixed with virus and plated onto gelatinized wells of 12-well plates. Polybrene (Yeasen, Cat#40804ES76) was added at a concentration of 8 µg/ml. Cells were incubated with the virus for 24 h, fed with fresh media, and then incubated for an additional 24 h for recovery. All shRNA knockdown (KD) experiments concluded after a 5-day drug selection period (puromycin, 1 μg/ml).

### RNA isolation, RT-PCR and quantitative real-time PCR (qRT-PCR)

Total RNA was extracted from cells using TRIzol regent (Vazyme, Cat#R401-01) according to the manufacturer’s instructions. cDNAs were synthesized using Hifair^®^ III 1st Strand cDNA Synthesis Kit (Yeasen, Cat#11139ES60) from 2 μg of total RNA. Low-cycle PCR for detecting patterns of alternative splicing was subsequently performed with GoldenStar^®^ T6 Super PCR Mix (1.1 ×) (TSINGKE, Cat#TSE101) [25]. Primers sequences are listed in Additional file 1: Table S1. *Gapdh* was used as control. PCR products were analyzed on 1% agrose gel and visualized using a Tanon 2500 Fully Automated Digital Gel Imager (Tanon) and quantified using the QuantStudio 3 Real-Time PCR System (ThermoFisher Scientific). For qPCR, Hieff UNICON^®^ Universal Blue qPCR SYBR Green Master Mix (Yeasen, Cat#11184ES08) was used following the manufacturer’s instructions. Primer sequences used for RT-PCR and qRT-PCR are listed in Additional file 1: Table S1.

### Alkaline phosphatase (ALP) assay

After PBS buffer washing, wild-type (WT), RA-treated and shRNA KD E14 ESCs were collected in cell lysis buffer (20 mM Tris-Cl, pH 7.5, 150 mM NaCl, 1% Triton X-100). The harvested cells underwent sonication and subsequent centrifugation at 12,000 rpm for 20 min at 4 °C. The resulting supernatant was utilized for ALP activity assessments using Alkaline Phosphatase Assay Kit (Beyotime, Cat#P0321S). ALP activity was measured three times with the same sample as the manufacturer's instructions using BioTek Epoch Microplate Spectrophotometer (Agilent).

### Chromatin immunoprecipitation (ChIP)

The experiment was conducted following the protocol described [26]. Approximately 1 × 10^7^ WT or *hnRNPK*-KD E14 ESCs were crosslinked with 1% formaldehyde for 10 min at room temperature (RT) and subsequently lysed successively with Lysis Buffer 1 (50 mM HEPES-KOH, pH 7.5, 140 mM NaCl, 1 mM EDTA, 10% glycerol, 0.5% NP-40, 0.25% Triton X-100), Lysis Buffer 2 (10 mM Tris-HCl, pH 8.0, 200 mM NaCl, 1 mM EDTA, 0.5 mM EGTA) and Lysis Buffer 3 (10 mM Tris-HCl, pH 8.0, 100 mM NaCl, 1 mM EDTA, 0.5 mM EGTA, 0.1% Na-Deoxycholate, 0.5% N-lauroylsarcosine, 1 × protease inhibitor). The cell suspension was sonicated using a Covaris M220 Focused-ultrasonicator (water temperature 4 °C, incident power 75 W, duty factor 10) for 5 min to yield DNA fragments approximately 200-300 bp in size. After centrifugation at 20,000 g for 10 min at 4 °C, the supernatant was incubated with 3 ug GST-antibody (Proteintech, Cat#075501) overnight at 4 °C with slow rotation. The next day, 20 ul Pierce™ Protein A/G Magnetic Beads (ThermoFisher Scientific, Cat#26162) were added into the supernatants and rotated at 4 °C for 2 h. The beads were then washed successively with high salt buffer (20 mM Tris-HCl, pH 8.0, 500 mM NaCl, 2 mM EDTA, 1% Triton X-100, 0.1% SDS), LiCl buffer (10mM Tris-HCl, pH 8.0, 250 mM LiCl, 1 mM EDTA, 1% Na-Deoxycholate, 1% NP-40) and TE buffer (10 mM Tris-HCl, pH 8.0, 1 mM EDTA). The protein-DNA complexes were eluted with elution buffer (50 mM Tris-HCl, pH 8.0, 1 mM EDTA, 1% SDS) at 65 °C for 30 min. The co-eluted RNA was removed by RNase A (Beyotime, Cat# ST577) treatment at 37 °C for 2 h, followed by protein cleanup using Proteinase K (Beyotime, Cat# ST533) at 55 °C for 2 h. DNA fragments were extracted and precipitated in isopropanol for subsequent qRT-PCR analyses.

### Co-immunoprecipitation (Co-IP)

Around 1 × 10^7^ WT E14 ESCs were lysed in IP lysis buffer (50 mM Tris-HCl, pH 7.5, 150 mM NaCl, 1% Triton X-100, 1 mM EDTA, 10% glycerol, 1 × protease inhibitor cocktail (MedChemExpress, Cat#HY-K0010), 1 mM VRC (Beyotime, Cat#R0108)) on ice for 1 h. Lysates were homogenized with a 0.4 mm needle and centrifuged at 12,000 rpm for 20 min at 4 °C. Supernatants were incubated with anti-SOX2 antibody (Cell Signaling, Cat#23064) or IgG (Solarbio, Cat#SP034) and rotated overnight at 4 °C. On the following day, 20 ul Pierce™ Protein A/G Magnetic Beads (ThermoFisher Scientific, Cat#26162) were added into the supernatants and rotated at 4 °C for 2 h. The beads were washed four times with wash buffer (50 mM Tris-HCl, pH 7.5, 150 mM NaCl, 0.5% NP-40, 1 × protease inhibitor cocktail (MedChemExpress, Cat#HY-K0010), 1 mM VRC (Beyotime, Cat#R0108)) and eluted by boiling in 50 μl SDS loading buffer, followed by Western blotting with the relevant antibodies (anti-hnRNPK, Santa Cruz Biotechnology, Cat#sc-28380; anti-HEXIM1, proteintech, Cat#15,676-1-AP; anti-CYCLINT1, abcam, Cat#ab184703; anti-SOX2, Cell Signaling technology, Cat#23064S).

### Pull-down assay

Mouse *Sox2* (Gene ID: 20674), *Sox2*-HMG, *Sox2*-ΔHMG, *hnRNPK* (Gene ID: 15387), *Hexim1* (Gene ID: 192231), and *CyclinT1* (Gene ID: 12455) cDNAs were amplified using 2 × Phanta Max Master Mix (Vazyme, Cat#P515-01). Expression plasmids for GST-tagged proteins (SOX2, SOX2-HMG, SOX2-ΔHMG and HEXIM1) were created by cloning the cDNAs into the PGEX-4T-1 vector at the EcoRI-XhoI sites. The expression plasmid for His-tagged hnRNPK was constructed by cloning its cDNA into pET28a at the NcoI-XhoI sites. The expression plasmid for His-tagged hnRNPK-ΔKI was constructed by PCR amplification of the His-tagged hnRNPK expression plasmid, followed by blunt-end ligation using the Blunt/TA Ligase Master Mix (NEB, Cat#M0367). The expression plasmid for HA-tagged SOX2 was generated by ligating the HA-tag DNA fragment at the 3’ end of the *Sox2* cDNA and cloning it into the pET28a vector at the NcoI-XhoI sites. The expression plasmid for Flag-tagged CYCLINT1 was constructed by ligating the Flag-tag DNA fragment at the 3’ end of the *CyclinT1* cDNA and cloning it into the pET28a vector at the NcoI-XhoI sites. The primers used are detailed in Additional file 1: Table S1. *E. coli* BL21 (DE3) cells transformed with the constructs were cultivated at 37 °C until reaching OD_600_ = 0.4-0.6, and protein expression was induced with 0.2 mM IPTG for SOX2, SOX2-HMG, SOX2-ΔHMG, hnRNPK, hnRNPK-ΔKI and CYCLINT1 at 30 °C for 4 h, or 0.2 mM IPTG for HEXIM1 overnight at 20 °C. Cells were harvested by centrifugation, resuspended in buffer A (50 mM Tris-HCl, pH 8.0, 150 mM NaCl, 1% NP-40, 1 mM EDTA, 1 × protease inhibitor cocktail (MedChemExpress, Cat#HY-K0010)), and incubated on ice for 30 min. After centrifugation at 12,000 g for 30 min at 4 °C, supernatants were incubated with glutathione agarose (Sangon biotech, Cat#C600031) or Ni-NTA agarose (Biomed, Cat# PA304-01) overnight at 4 °C with rotation and washed four times with buffer A. Proteins bound to glutathione beads were eluted with buffer B (50 mM Tris-HCl, pH 8.0, 150 mM NaCl, 1% NP-40, 1 mM EDTA, 15 mM reduced glutathione), separated on a 12% SDS-PAGE gel, and assayed by Western blot using antibodies (anti-His, Abcam, Cat#ab18184; anti-GST, Santa Cruz Biotechnology, Cat#sc-138).

### Immunofluorescence staining (IF)

Mouse *Sox2* (Gene ID: 20674) and *hnRNPK* (Gene ID: 15387) cDNAs were amplified using 2 × Phanta Max Master Mix (Vazyme, Cat#P515-01). The expression plasmid for HA-tagged SOX2 was created by ligating the HA-tag DNA fragment at the 3’ end of the *Sox2* cDNA and then cloned into the pcDNA3.1 vector at the NheI-XbaI sites. The expression plasmid for Flag-tagged hnRNPK was constructed by ligating the Flag-tag DNA fragment at the 3’ end of the *hnRNPK* cDNA and subsequently cloned into the pcDNA3.1 vector at the NheI-XhoI sites. The expression plasmids were then co-transfected or independently transfected into NIH3T3 cells using Lipofectamine 3000 (Yeasen, Cat#40802ES03) following the manufacturer’s instructions. NIH3T3 cells expressing HA-tagged SOX2 and/or Flag-tagged hnRNPK were fixed with 4% paraformaldehyde (LEAGENE, Cat#DF0135) for 15 min. Subsequently, they were permeabilized with 0.1% Triton X-100 for 10 min, blocked with 3% sheep serum (Sangon biotech, Cat#E510009) for 1 h at RT, and incubated with the appropriate antibodies (anti-Flag, proteintech, Cat#66008–4-Ig; anti-HA, proteintech, Cat#81290–1-RR) overnight at 4 °C. Following six washes with PBST, the cells were stained with secondary fluorescent antibodies (Goat anti-Rabbit IgG (H + L), Invitrogen, Cat#A11008; Goat anti-Mouse IgG (H + L), Invitrogen, Cat#A-11005) for 2 h at RT and then washed. The immuno-stained cells were observed using a Nikon ECLIPSE Ti2-E confocal microscope (Nikon) equipped with DAPI, GFP and RFP filter cubes.

### Electrophoretic mobility shift assay (EMSA)

DNA binding reactions were conducted following established protocols [25]. Subsequently, the reaction samples were separated on an 8% native polyacrylamide gel in darkness at 4 °C in 0.5 × TAE at 200 V. Signal visualization was performed using a Typhoon™ FLA 7000 biomolecular imager (GE Healthcare), and quantification was carried out using the ImageQuant TL software (GE Healthcare).

### Dual-luciferase reporter assay

The enhancer region of *Fgf4* (mm10, chr7:144,864,284–144,864,588) was cloned by PCR amplification of genomic DNA from C57BL/6 mice, and subsequently inserted into the PGL3-promoter vector with KpnI and NheI restriction sites to generate the plasmid for luciferase activity assay. The sequence and primers used are detailed in Additional file 1: Table S1. WT or *hnRNPK*-KD E14 ESCs were seeded into 12-well plates at a density of 0.5 × 10^6^ cells per well. Luciferase assay was performed in ESCs transfected with 10 ng of pRL-TK and 200 ng of luciferase reporter plasmids containing the *Fgf4* enhancer for 48 h. Afterward, the cells were lysed, and luciferase activity was assessed using a Dual-Luciferase Reporter assay kit (Yeasen, Cat#11402ES60) following the manufacturer’s instructions. The measurements were conducted using triplicate biological samples.

### Preparation of hnRNPK and HEXIM1 proteins

The proteins were expressed in *E. coli* BL21 (DE3). Transformed cells were cultivated at 37 °C until reaching OD_600_ = 0.4–0.6, and protein expression was induced with 0.2 mM IPTG for hnRNPK at 30 °C for 4 h or 0.2 mM IPTG for HEXIM1 overnight at 20 °C. Cells were then collected by centrifugation, resuspended in lysis buffer 1 (50 mM Tris-HCl, pH 7.4, 500 mM NaCl, 20 mM imidazole, 6% glycerol, 1 mM DTT, 1 mM PMSF) for hnRNPK or lysis buffer 2 (50 mM Tris-HCl, pH 7.4, 500 mM NaCl, 5 mM MgCl_2_, 20 mM imidazole, 6% glycerol, 1 mM DTT, 1 mM PMSF) for HEXIM1, and disrupted by ultrasonication (Xinzhi JY92 Ultrasonic homogenizer; 300 W, 30 min for a 30 ml suspension with on/off cycles of 3 s/6 s). The proteins were captured with Ni-NTA resin (ThermoFisher Scientific, Cat#88222) or glutathione agarose (Sangon biotech, Cat#C600031). Purified proteins were subsequently dialyzed into lysis buffer using ultrafiltration tubes (Merck, Cat#UFC900308). Protein concentrations were determined by measuring the UV absorbance at 280 nm.

### Preparation of RNA substrates

The mouse 7SK snRNA gene (Gene ID: 19817) and its truncation SL1 were amplified with the primers indicated in Additional file 1: Table S1 and in vitro transcriptions were performed using TranscriptAid T7 High Yield Transcription Kit (ThermoFisher Scientific, Cat#K0441) following the manufacturer’s instructions. After phenol–chloroform extraction and ethanol precipitation, RNA concentrations were determined by measuring the UV absorbance at 260 nm and analyzed on 2% agarose gels.

### RNA immunoprecipitation assay (RIP)

In vitro RIP was performed with purified His-tagged hnRNPK and GST-tagged HEXIM1. After washing with RNA binding buffer (50 mM Tris-HCl, pH 8.3, 135 mM KCl, 15 mM NaCl, 10% glycerol) for three times, 50 μl Ni-NTA magnetic resin (Biomed, Cat#PA303) or 50 μl glutathione agarose (Sangon biotech, Cat#C600031) was incubated with incubate with 1.2 nmol His-tagged hnRNPK or 1.2 nmol GST-tagged HEXIM1 and varying amounts of His-tagged hnRNPK with gentle rotating at 4 °C for 2 h. After three additional washes with RNA binding buffer, 1.2 nmol RNAs were combined with the beads and incubated with gentle rotation at 4 °C for 2 h. Following four washes with RNA binding buffer, the bound RNAs were eluted with 10 mM EDTA, pH 8.2, and 95% formamide at 90 °C for 10 min. The elution fractions were then separated on a 2% agarose gel in 1 × TAE at 200 V.

RIP using cell lysate was conducted as previously described [27]. Briefly, approximately 2 × 10^7^ E14 ESCs were detached with 0.25% Trypsin (Gibco, Cat#25200114). The cell pellet was resuspended in an equal volume of lysis buffer (100 mM KCl, 5 mM MgCl_2_, 10 mM HEPES, pH 7.0, 0.5% NP40, 1 mM DTT, 100 U/ml RNase Out (MedChemExpress, Cat#HY-K1033), 400 μM VRC (Beyotime, Cat#R0108), Protease inhibitor cocktail (MedChemExpress, Cat#HY-K0010)), kept on ice for 5 min, and then immediately used for immunoprecipitation or transferred to liquid nitrogen for storage. The lysate was thawed on ice, and cell debris were removed by centrifugation at 4 °C. Prior to immunoprecipitation, the lysate was pre-cleared with Pierce™ Protein A/G Magnetic Beads (ThermoFisher Scientific, Cat#26162) and then subjected to the addition of anti-GST antibodies (proteintech, Cat#10000-0-AP) pre-bound to Pierce™ Protein A/G Magnetic Beads (ThermoFisher Scientific, Cat#26162) for 4 h at 4 °C. In total, 5 μg of antibodies were used for each RIP reaction. Beads were then washed five times in ice-cold NT2 buffer (50 mM Tris-HCl, pH 7.4, 150 mM NaCl, 1 mM MgCl_2_, 0.05% NP40). RNAs were released from ribonucleoprotein complexes with Proteinase K (Beyotime, Cat#ST533) at 55 °C for 30 min. RNA was isolated with TRIzol reagent (Vazyme, Cat#R401-01) and precipitated in 75% ethanol, resuspended in DEPC H_2_O for further qRT-PCR analysis. Primer sequences used are listed in Additional file 1: Table S1.

### RNA-sequencing (RNA-seq)

RNA-seq was performed in E14 ESCs infected with control shRNA, *Sox2* shRNAs, *hnRNPK* shRNAs or *Cdk9* shRNAs. Biological duplicates were prepared. Total RNA from each sample was extracted from the cells with TRIzol regent (Vazyme, Cat#R401-01). The library construction and sequencing were performed at BGI with a BGISEQ platform with 150-bp paired-end reads.

### Bioinformatics analysis

The RNA-seq reads underwent initial processing by trimming sequencing adapters and filtering out read pairs with low quality or complexity using Trimmomatic [28]. The resulting clean read pairs were then aligned to GENCODE vM23 (mm10) using STAR (version 2.7.10) [29]. Significantly differential transcript expression was determined using edgeR [30] with q value < 0.05 and absolute log_2_ fold change (log_2_FC) value > 0.6. Differential splicing analysis was performed using rMATS (v4.0.1) [31] based on GENCODE vM23 transcript models, with a false discovery rate (FDR) threshold set at 0.05. Following the identification of alternative splicing events by rMATS, those with an FDR < 0.05 were aggregated within the same cluster for downstream analysis. Each specific type of alternative splicing event is examined separately. For A3SS or A5SS alternative splicing events, the datasets were aligned using common gene ID, gene symbol, short and long exon information, as well as flanking exon details. Concerning the other three types (SE, RI, MXE), shared alternative splicing events are identified by aligning the start and end positions of upstream and downstream exons. The number of alternative splicing events or genes in different alternative splicing types was recorded and plotted with the Venn function in jvenn (https://www.bioinformatics.com.cn/static/others/jvenn/example.html). For the analysis of alternative splicing junctions, junction events were categorized based on strand information, and the exon–intron junction positions were aligned to GENCODE vM23 (mm10) with a 25 bp extension both upstream and downstream of the junction position. Reads not adhering to the specified junction criteria or not meeting the FDR < 0.1 criteria were excluded. Subsequently, a probability calculation was conducted, and a position weight matrix (PWM) was generated to derive motifs. The isoform switch analysis was performed by IsoformSwitchAnalyzeR [32].

### Rescue experiment

The *hnRNPK* (Gene ID: 15387) was amplified using 2 × Phanta Max Master Mix (Vazyme, Cat#P515-01). The expression plasmid for Flag-tagged hnRNPK was constructed by ligating the Flag-tag DNA fragment at the 3’ end of the hnRNPK cDNA and subsequently cloned into the pcDNA3.1 vector at the NheI-XhoI sites. The expression plasmid for Flag-tagged hnRNPK-ΔKI was constructed by PCR amplification of the Flag-tagged hnRNPK expression plasmid, followed by blunt-end ligation using the Blunt/TA Ligase Master Mix (NEB, Cat#M0367). The expression plasmid was then transfected into *hnRNPK*-KD E14 ESCs using Lipofectamine 3000 (Yeasen, Cat#40802ES03) following the manufacturer’s instructions. Primers sequences used for the plasmid construction are listed in Additional file 1: Table S1. After 48 h of cultivation, cells were harvested to for test.

The *Eif4a2*^*PTC*^ (Gene ID: 13682) and *Ash2l-b* (Gene ID: 23808) cDNAs were amplified using 2 × Phanta Max Master Mix (Vazyme, Cat#P515-01) and then inserted into the pLVX-TRE3G vector (Takara, Cat#631187) using NdeI and EcoRI restriction sites, respectively, to generate plasmids for lentiviral particle production. Primers sequences used for the plasmid construction are listed in Additional file 1: Table S1. Following the manufacturer’s instructions, *hnRNPK*-KD and *Sox2*-KD E14 ESCs were infected with lentivirus, and 4 ug/ml doxycycline (Dox) was added 24 h post-infection. After 48 or 72 h of cultivation, cells were harvested to assess the rescue effects.

### Availability of data and materials

All raw sequence data and processed results for bioinformatics analysis have been deposited in the NCBI Gene Expression Omnibus (GEO) Archive under the accession number GSE232524. All data used in this study are available from the corresponding author on reasonable request.

## Results

### SOX2 and hnRNPK are both required for maintaining pluripotency in mouse embryonic stem cells

**Fig. 1:**
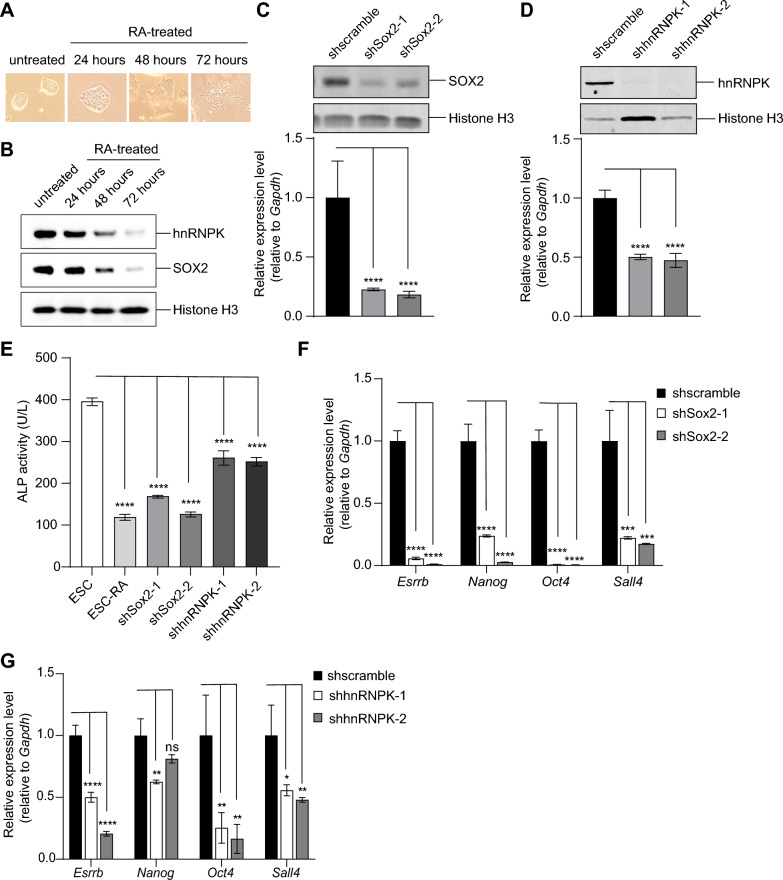
hnRNPK and SOX2 are both indispensable for maintaining pluripotency in mESCs. **A** Morphology of E14 ESCs before and after treatment with RA for 24 h, 48 h or 72 h. **B** Western blot analysis of hnRNPK, SOX2 and Histone H3 protein levels in the nuclear chromatin preparation isolated from E14 ESCs from (**A**). **C** Western blot (upper panel) and qRT-PCR (lower panel) analyses of SOX2 protein and RNA expression levels in *Sox2*-KD E14 ESCs. shscramble: E14 ESCs infected with scrambled control shRNA; shSox2-1 and shSox2-2: E14 ESCs infected with shRNAs targeting *Sox2*. **D** Western blot (upper panel) and qRT-PCR (lower panel) analyses of hnRNPK protein and RNA expression levels in *hnRNPK*-KD E14 ESCs. shscramble: E14 ESCs infected with scrambled control shRNA; shhnRNPK-1 and shhnRNPK-2: E14 ESCs infected with shRNAs targeting *hnRNPK*. **E** The expression of ALP activity in WT (ESC), RA-treated (ESC-RA), *Sox2*-KD (shSox2-1 and shSox2-2) and *hnRNPK*-KD (shhnRNPK-1 and shhnRNPK-2) E14 ESCs. **F** The relative transcript levels of selected pluripotency markers in *Sox2*-KD E14 ESCs were analyzed by qRT-PCR. **G** The relative transcript levels of selected pluripotency markers in *hnRNPK*-KD E14 ESCs were analyzed by qRT-PCR. Data in Fig. 1 are represented as mean ± SD (n = 3). ANOVA was used to assess significance (****, ***, ** and * indicate P-values of < 0.0001, < 0.001, < 0.01 and < 0.05, respectively; ns indicates *P*-value = 0.0507)

It has been shown that thousands of hnRNPK target sites are often co-occupied by pluripotency-related factors, such as SOX2, in mouse embryonic stem cells (mESCs) [22]. To explore the correlation between the pluripotent state and the co-localization of SOX2 and hnRNPK, we induced differentiation in E14 ESCs using retinoic acid (RA) and assessed the chromatin binding of SOX2 and hnRNPK. Chromatin was extracted from ESC nuclei, and the levels of SOX2 and hnRNPK in the chromatin preparation were compared. The results revealed robust chromatin binding of both SOX2 and hnRNPK in undifferentiated ESCs, while their chromatin binding significantly decreased in RA-treated differentiated ESCs (Fig. 1A, B).

To further assess the significance of SOX2 and hnRNPK in maintaining pluripotency, we employed shRNAs targeting their respective transcripts to downregulate their expression in E14 ESCs. Successful knockdown (KD) of both *Sox2* and *hnRNPK* was confirmed through Western blot and qRT-PCR analyses (Fig. 1C, D). ESC pluripotency was evaluated by measuring alkaline phosphatase (ALP) enzymatic activity. Similar to ESCs treated with RA for 72 h (ESC-RA), ESCs with *Sox2* or *hnRNPK* KD (shSox2-1, shSox2-2; shhnRNPK-1, shhnRNPK-2) exhibited significantly reduced ALP activity compared to wild-type (WT) ESCs (ESC) (Fig. 1E). Consistently, KD of *Sox2* or *hnRNPK* in ESCs resulted in the suppression of mRNA expression levels of pluripotency-related genes, including *Esrrb*, *Nanog*, *Oct4*, and *Sall4* (Fig. 1F, G). Additionally, the expression of both *Sox2* and *hnRNPK* was downregulated in either *Sox2*-KD or *hnRNPK*-KD ESCs (Additional file 2: Figure S1), suggesting a mutual dependency between them. In summary, these findings underscore the necessity of the concurrent presence of hnRNPK and SOX2 in maintaining pluripotency.

### SOX2 directly associates with hnRNPK

**Fig. 2:**
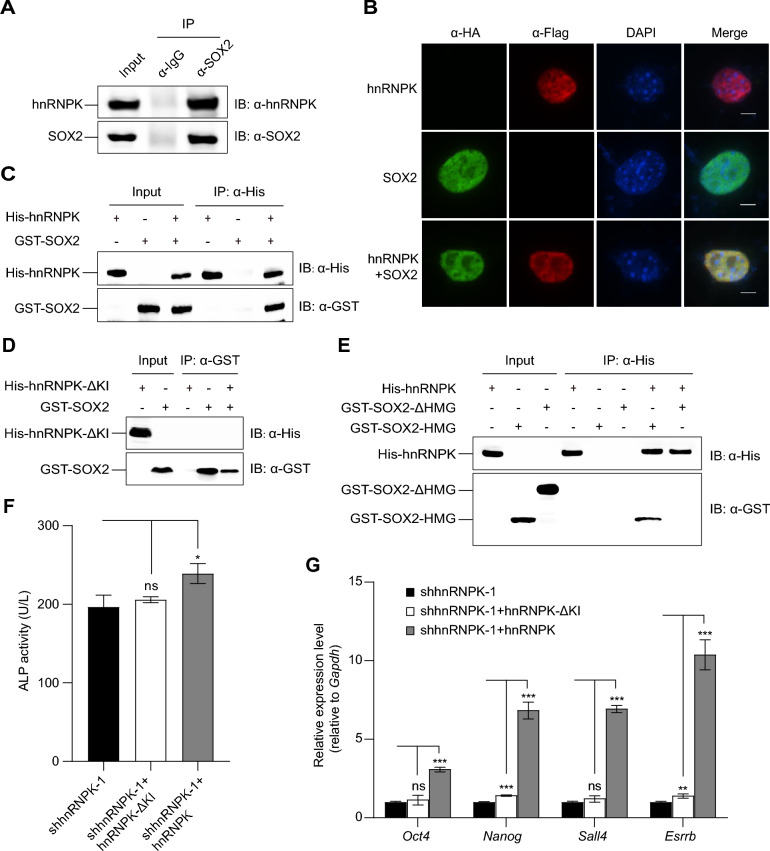
SOX2 directly interacts with hnRNPK. **A** Co-IP of SOX2 with hnRNPK. Whole cell lysates of E14 ESCs were immunoprecipitated with anti-SOX2 antibody or IgG. IP products were blotted with anti-SOX2 and anti-hnRNPK antibodies. **B** Immunofluorescence colocalization of SOX2 and hnRNPK. NIH3T3 cells expressing HA-SOX2 and/or Flag-hnRNPK were immuno-stained for HA (green) and Flag (red). Colocalization of SOX2 and hnRNPK was indicated in merged panels (yellow). DAPI stained cell nuclei (blue). Scale bars: 10 μm. **C** *In vitro* pull-down assays of His-hnRNPK with GST-SOX2. *E. coli* lysates expressing GST-SOX2 were incubated with either His-hnRNPK or His coupled with Ni-NTA agarose beads, and the resulting pull-down products were detected using anti-His or anti-GST antibodies. **D** *In vitro* pull-down assays of His-hnRNPK-ΔKI with GST-SOX2. *E. coli* lysates expressing His-hnRNPK-ΔKI derivates were incubated with either GST-SOX2 or GST coupled with glutathione agarose beads, and the resulting pull-down products were detected using anti-His or anti-GST antibodies. **E** *In vitro* pull-down assays of His-hnRNPK with GST-SOX2 derivates. *E. coli* lysates expressing GST-SOX2 derivates were incubated with either His-hnRNPK or His coupled with Ni-NTA agarose beads, and the resulting pull-down products were detected using anti-His or anti-GST antibodies. The symbols ‘ + ’ and ‘-’ denote the presence and absence, respectively, of the corresponding protein in the reaction mix. **F** The expression of ALP activity in *hnRNPK*-KD E14 ESCs (shhnRNPK-1) and *hnRNPK*-KD E14 ESCs expressed hnRNPK (shhnRNPK-1 + hnRNPK) or hnRNPK-ΔKI (shhnRNPK-1 + hnRNPK-ΔKI). **G** The relative transcript levels of selected pluripotency markers in E14 ESCs from (**F**) were analyzed by qRT-PCR. Data are represented as mean ± SD (n = 3). ANOVA was used to assess significance (***, ** and * indicate P-values of < 0.001, < 0.01 and < 0.05, respectively; ns indicates not significant)

Our previous study demonstrated the co-presence of SOX2 and hnRNPK within a common interactome during mouse embryonic fibroblasts (MEF) reprogramming [9]. Here, we validated their association using an anti-SOX2 immunoprecipitation (IP) assay followed by Western blot in E14 ESCs. Western blot analysis confirmed the co-immunoprecipitation (co-IP) of SOX2 with hnRNPK, indicating that SOX2 and hnRNPK are components of the same complex within the pluripotent background (Fig. 2A). This colocalization was further affirmed through immunofluorescence staining (IF). NIH3T3 cells expressing HA-tagged SOX2 (HA-SOX2) and Flag-tagged hnRNPK (Flag-hnRNPK) were fixed 48 h post-transfection and subjected to primary antibody against HA (derived from rabbit) or/and Flag (derived from mouse). Confocal microscopy analysis of the IF assay revealed the close proximity of SOX2 and hnRNPK within the nuclear region (Fig. 2B).

The colocalization of SOX2 and hnRNPK prompted us to investigate their potential direct interaction. To examine this, we expressed recombinant glutathione-S-transferase (GST)-tagged SOX2 (GST-SOX2) and His-tagged hnRNPK (His-hnRNPK) in *E. coli* (BL21) cells and conducted a pull-down assay using bacterial extracts. As confirmed by subsequent Western blot analysis, SOX2 was found to directly associate with hnRNPK (Fig. 2C). hnRNPK interacts with over 100 protein partners through its K-protein-interaction (KI) domain, enabling its participation in crucial cellular processes such as DNA transcription, RNA splicing, RNA stability, and translation [33]. To investigate the role of the KI domain in the interaction between hnRNPK and SOX2, we generated an expression plasmid for His-tagged truncated hnRNPK lacking the KI domain (hnRNPK-ΔKI). Recombinant GST-tagged SOX2 protein (GST-SOX2) and His-tagged hnRNPK-ΔKI protein (His-hnRNPK-ΔKI) were expressed in *E. coli* (BL21). *In vitro* pull-down assay using bacterial lysates, followed by Western blot analysis, demonstrated that the absence of the KI domain in hnRNPK disrupted its interaction with SOX2, thus indicating that the KI domain mediates hnRNPK’s interaction with SOX2 (Fig. 2D). Furthermore, to identify the specific domain(s) of SOX2 responsible for its interaction with hnRNPK, we created expression plasmids for GST-tagged truncated SOX2 variants: SOX2-HMG (40–119, containing the HMG domain) and SOX2-ΔHMG (lacking 40–119, devoid of the HMG domain). These truncated SOX2 proteins and His-hnRNPK recombinant protein were expressed in *E. coli* (BL21). The subsequent *in vitro *pull-down assay using bacterial lysates, combined with Western blot analysis, revealed that SOX2 interacts with hnRNPK through its HMG domain (Fig. 2E), suggesting that hnRNPK may have an impact on SOX2-mediated transcriptional regulation. Partial dysfunction of hnRNPK is likely due to the loss of protein-protein interaction [33–35]. To assess the significance of hnRNPK-SOX2 interaction in maintaining pluripotency in ESCs, we evaluated ALP activity and the expression of pluripotency-related genes in *hnRNPK*-KD E14 ESCs upon expression of hnRNPK or hnRNPK-ΔKI. We found that hnRNPK expression increased both ALP activity and the expression of pluripotency-related genes, whereas expression of hnRNPK-ΔKI, which cannot form a complex with SOX2, showed no significant effect (Fig. 2F, G), highlighting the importance of hnRNPK-SOX2 interaction in maintaining ESC pluripotency.

### hnRNPK is dispensable for SOX2 in transcriptional regulation

**Fig. 3:**
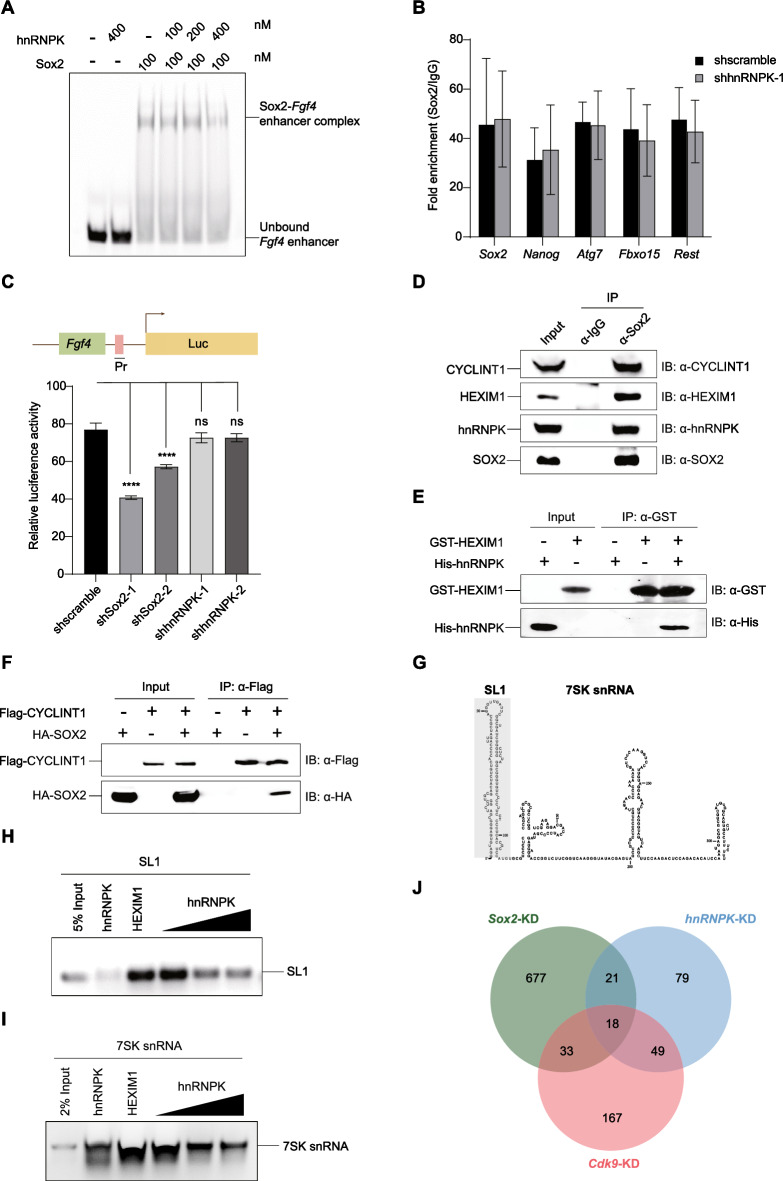
hnRNPK has little role in SOX2 transcriptional regulation by facilitating transcription elongation. **A** EMSA of Cy5-labeled *Fgf4* enhancer DNA (50 nM) with SOX2 (100 nM) and increasing concentrations (100–400 nM) of hnRNPK. Concentrations of SOX2 and hnRNPK are labelled above each lane. Free DNA and protein-DNA complex are marked. ‘-’ denotes the absence of the corresponding protein in the reaction mix. **B** ChIP analysis for SOX2 in E14 ESCs with or without *hnRNPK* KD. Relative enrichments to IgG-ChIP control are shown. **C** A dual-luciferase reporter assay validated hnRNPK’s impact on the transcriptional activation of SOX2 via an *Fgf4* enhancer-containing reporter plasmid. Schematic showing luciferase reporter with *Fgf4* enhancer region containing SOX2 binding motif (upper panel) and relative expression of luciferase in control (shscramble), *Sox2*-KD (shSox2-1 and shSox2-2) and *hnRNPK*-KD (shhnRNPK-1 and shhnRNPK-2) E14 ESCs (lower panel). Data are represented as mean ± SD (n = 3). ANOVA was used to assess significance (**** indicates *P*-value of < 0.0001; ns indicates not significant). Pr: SV40 promoter. **D** Co-IP analysis verifying interactions between SOX2, hnRNPK, HEXIM1, and CYCLINT1. Endogenous SOX2 was immunoprecipitated from E14 ESCs whole cell lysate using anti-SOX2 antibody or IgG. IP products were blotted with anti-Sox2, anti-hnRNPK, anti-HEXIM1 and anti-CYCLINT1 antibodies. **E** *In vitro* pull-down assays of GST-HEXIM1 with His-hnRNPK. *E. coli* lysates expressing His-hnRNPK were incubated with either GST-HEXIM1 or GST coupled with glutathione sepharose beads, and the resulting pull-down products were detected using anti-His or anti-GST antibodies. The symbols ‘ + ’ and ‘-’ denote the presence and absence, respectively, of the corresponding protein in the reaction mix. **F** Co-IP of SOX2 and CYCLINT1. Whole cell lysates of *E. coli* expressing HA-SOX2 and/or Flag-CYCLINT1 were immunoprecipitated with anti-Flag antibody. IP products were blotted with anti-HA and anti-Flag antibodies. **G** Scheme of 7SK snRNA and its 5’ stem loop (SL1). The sequence of SL1 used is highlighted. **H-I.** *In vitro* RIP with 1.2 nmol HEXIM1 and 1.2 nmol 7SK snRNA constructs with increasing amount of hnRNPK. The names of proteins and RNA substrates are as indicated above each panel. **J** Analysis of differentially expressed genes obtained through RNA-seq. The Venn diagram displays the number of genes that exhibit differential expression in *Sox2*-KD, *hnRNPK*-KD, and *Cdk9*-KD E14 ESCs compared to control (shscramble) E14 ESCs

We next sought to understand how the SOX2/hnRNPK complex contributes to the maintenance of pluripotency. Given the pivotal role of SOX2 as a transcription factor in ESC maintenance, we investigated whether hnRNPK influences SOX2 in transcriptional regulation. We initially examined the effect of hnRNPK on SOX2’s DNA-binding activity using electrophoretic mobility shift assays (EMSAs) with a Cy5-labeled DNA of the *Fgf4* enhancer element. Interestingly, increasing the quantity of hnRNPK does not affect the binding of SOX2 to the DNA substrate (Fig. 3A). Consistently, ChIP-qPCR assays also revealed that KD of *hnRNPK* did not disrupt the binding of SOX2 to its target genes (Fig. 3B). To further evaluate the influence of hnRNPK on SOX2-mediated transcriptional regulation, we conducted a dual-luciferase reporter assay with a SOX2-targeted *Fgf4* enhancer construct (Fig. 3C, upper panel) transiently transfected into E14 ESCs. In cells expressing *Sox2*-shRNAs, the expression of the reporter gene was significantly reduced, while it showed minimal change in cells expressing *hnRNPK*-shRNAs (Fig. 3C, lower panel). These results suggest that hnRNPK does not impact SOX2 in transcriptional regulation by influencing the interaction of SOX2 with its target *cis*-regulatory element.

hnRNPK was proposed to have roles in RNA polymerase II (Pol II)-mediated transcription elongation due to its associations with transcription factors, transcription machinery, and chromatin-remodeling complexes [36]. Transcription elongation is stimulated by the positive transcription elongation factor B (P-TEFb, primarily composed of CYCLINT1 and CDK9), which is suppressed within the 7SK small nuclear ribonucleoprotein (7SK snRNP, composed of 7SK snRNA, MePCE, and Larp7). The RNA‐binding domain of HEXIM1 interacts with 7SK snRNA, and P-TEFb enters 7SK snRNP through association with HEXIM1. Efficient transcriptional elongation for many cellular mRNAs requires transcription factors that disengage P-TEFb from the 7SK snRNP by reducing the interaction between HEXIM and 7SK snRNA [37]. We hypothesized that hnRNPK could play a role in attenuating the association between 7SK snRNA and HEXIM1, facilitating the release of the P-TEFb from the 7SK snRNP and thereby promoting SOX2-mediated transcriptional elongation. To explore this hypothesis, we first examined the interaction between the SOX2/hnRNPK complex and 7SK snRNP through an anti-SOX2 co-IP assay with E14 ESCs lysate. We observed that Sox2 interacts with hnRNPK, HEXIM1, and CYCLINT1 (Fig. 3D). To further investigate these interactions, we conducted pull-down assays using tagged proteins obtained from *E. coli* extracts and found a direct interaction between hnRNPK and HEXIM1 (Fig. 3E), while SOX2 associates with CYCLINT1 (Fig. 3F). HEXIM1 directly binds to the 5’ hairpin (SL1) of 7SK snRNA [38, 39]. We subsequently examined the impact of hnRNPK on the binding between HEXIM1 and 7SK snRNA, utilizing 7SK snRNA constructs (Fig. 3G) and purified hnRNPK or/and HEXIM1 in an RNA immunoprecipitation (RIP) assay. With increasing amounts of hnRNPK, it displaced the SL1 or the full-length 7SK snRNA from the preexisting HEXIM1/RNA complex (Fig. 3H, I), suggesting that hnRNPK’s interaction with 7SK snRNA and HEXIM1 might potentially impede the assembly of P-TEFb into the 7SK snRNP. To evaluate the role of hnRNPK in promoting SOX2 in transcriptional elongation, we isolated total RNA from E14 ESCs with KD of *hnRNPK*, *Sox2* or *Cdk9* and conducted RNA-sequencing (RNA-seq) analysis. Surprisingly, the data revealed that the expression of only 18 genes changed in all three groups (Fig. 3J). Collectively, these findings indicate that hnRNPK plays no evident biological role in SOX2 transcriptional regulation.

### hnRNPK cooperates with SOX2 in splicing

**Fig. 4:**
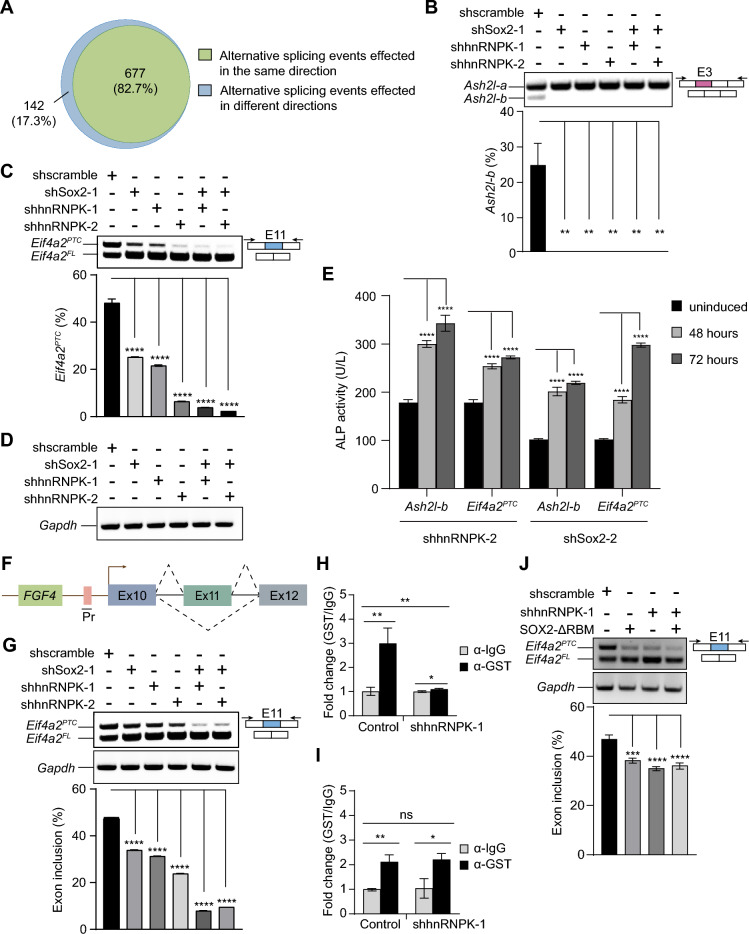
hnRNPK and SOX2 collaborate in the regulation of alternative splicing. **A** Venn diagrams showing the alternative splicing events affected by both hnRNPK and SOX2. **B**–**D** RT-PCR assays examining the splicing patterns of pluripotency-related genes *Ash2l* (**B**), *Eif4a2* (**C**), and the control gene *Gapdh* (**D**) in the control group (shscramble) of E14 ESCs and *Sox2*-KD (shSox2-1) or/and *hnRNPK*-KD (shhnRNPK-1, shhnRNPK-2) E14 ESCs. Histograms show quantifications of each RT-PCR measurement. **E** The expression of ALP activity in *hnRNPK*-KD (shhRNPK-2) and *Sox2*-KD (shSox2-2) E14 ESCs after 48 or 72 h of Dox-induced expression of *Ash2l-b* and *Eif4a2*^*PTC*^. **F**–**G** Eif4a2 minigene splicing assay validating the splicing pattern of *Eif4a2* transcript. Schematic representation of the Eif4a2 minigene (**F**). RT-PCR assays examining the splicing patterns of *Eif4a2* and the control gene *Gapdh* in the control group (shscramble) of E14 ESCs and *Sox2*-KD (shSox2-1) or/and *hnRNPK*-KD (shhnRNPK-1, shhnRNPK-2) E14 ESCs (**G**). The histogram shows quantifications of each RT-PCR measurement. **H**–**I.** RIP of GST-SOX2 (**H**) and GST-SOX2-ΔRBM (**I**) using anti-GST antibody with both WT and *hnRNPK*-KD E14 ESCs expressing GST-SOX2 or GST-SOX2-ΔRBM. RIP enrichment was measured by qRT–PCR, and values were normalized to background immunoprecipitation measured by isotype IgG. **J** Eif4a2 minigene splicing assay evaluating the impact of hnRNPK on SOX2-ΔRBM in *Eif4a2* transcript splicing. RT-PCR assays examining the splicing patterns of *Eif4a2* and the control gene *Gapdh* in the control group (shscramble) of E14 ESCs, E14 ESCs expressing GST-SOX2-ΔRBM (Sox2-ΔRBM), *hnRNPK*-KD E14 ESCs (shhnRNPK-1), and *hnRNPK*-KD E14 ESCs expressing GST-SOX2-ΔRBM. The histogram shows quantifications of each RT-PCR measurement. In this figure, error bars represent the mean ± SD from three biological replicates. Differences were compared using ANOVA (****, ***, ** and * indicate *P*-values of < 0.0001, < 0.001, < 0.01 and < 0.05, respectively; ns indicates not significant). Pr: SV40 promoter; Ex: exon

Besides of roles in transcriptional regulation, hnRNPK and SOX2 are also reported as splicing regulators that have roles in the regulation of alternative splicing events in cells [9, 19]. We quantified gene expression and analyzed isoform switching events in *Sox2*-KD and *hnRNPK*-KD E14 ESCs. Interestingly, we found a low correlation between the difference in isoform fraction (dIF) and the difference in gene expression (log2FC) (Additional file 2: Figure S2). This suggests that SOX2 and hnRNPK may regulate the switching of alternatively spliced isoforms independently of their roles in transcriptional regulation. Therefore, we analyzed the shared splicing events using rMATS for *hnRNPK*-KD E14 ESCs compared to *Sox2*-KD E14 ESCs. Remarkably, we identified 819 alternative splicing events in 301 genes that changed after the KD of either *hnRNPK* or *Sox2*. Among them, hnRNPK and SOX2 influenced 82.7% (677) of exclusion or inclusion events in the same direction (Fig. 4A, Additional file 1: Table S2), including a cluster of alternative splicing events in pluripotent genes. Further analysis of the PSI changes (ΔPSI) for these events revealed a strong correlation (R = 0.89, *P*-value < 2.2e-16) between splicing events after the KD of *hnRNPK* and those following the KD of *Sox2* (Additional file 2: Figure S3A), suggesting potential co-regulation of splicing by hnRNPK and SOX2.

Notably, within the top 10 genes exhibiting the highest occurrence of alternative splicing events among the 677 splicing events, we observed the presence of genes encoding two prominent regulators in maintaining pluripotency— the transcription factor ASH2L and the translation initiation factor eIF4A2 (Additional file 2: Figure S3B). ASH2L directly associates with super-enhancers of multiple stemness genes to modulate pluripotency and self-renewal in pluripotent stem cells [40]. It has two prominent splicing isoforms, *Ash2l-a* and *Ash2l-b*. Of these, *Ash2l-b* stands out as a prominent isoform in ESCs and holds crucial importance in somatic cell reprogramming and ESC maintenance [41]. In the control group of ESCs (shscramble), the *Ash2l-b* transcript level constitutes approximately 20% of the total *Ash2l* transcript (Fig. 4B), whereas in ESCs subjected to the KD of *hnRNPK* or/and *Sox2*, the *Ash2l-b* transcript became undetectable (Fig. 4B). eIF4A2 plays a critical role in maintaining the pluripotency of ESCs by regulating the translation of key factors [42, 43]. The *Eif4a2* gene generates two splice isoforms: one that produces a full-length protein (*Eif4a2*^*FL*^) (NM_013506.3) and another that is predicted to contain a premature termination codon (*Eif4a2*^*PTC*^) (NR_110335.1) [43]. Importantly, eIF4A2^PTC^, rather than eIF4A2^FL^, has a major impact on the exit from naïve pluripotency in mESCs. It elicits heightened mTORC1 activity and translation rates and causes ESC differentiation delays [43]. eIF4A2^PTC^ overexpression elevates transcript levels of naïve transcription factors *Klf4*, *Esrrb*, *Tfcp2l1*, and *Nanog* and an increased protein expression of NANOG [43, 44]. In the control group of ESCs (shscramble), *Eif4a2*^*PTC*^ transcript level constitutes approximately 50% of the total *Eif4a2* transcript (Fig. 4C). In contrast, in ESCs with KD of *hnRNPK* or *Sox2*, the *Eif4a2*^*PTC*^ transcript levels significantly decrease to around 20%-30% (Fig. 4C). Remarkably, the combined KD of *hnRNPK* and *Sox2* led to a drastic reduction in *Eif4a2*^*PTC*^ transcript levels, plummeting to less than 5% (Fig. 4C). As an extra control, the splicing of *Gapdh*, a target unrelated to both hnRNPK and SOX2, remained unaffected by the deficiency of hnRNPK and SOX2 (Fig. 4D). Furthermore, to confirm that the alternative splicing of these isoforms of *Ash2l* and *Eif4a2* were responsible to the pluripotency in ESCs, we created rescue constructs expressing *Ash2l-b* or *Eif4a2*^*PTC*^ and transfected them into *hnRNPK*-KD and *Sox2*-KD E14 ESCs. Following 48 and 72 h of induction with Dox, we observed a significant increase in ALP activity in both *hnRNPK*-KD and *Sox2*-KD E14 ESCs (Fig. 4E). In consistence, the mRNA expression levels of pluripotency-related genes, including *Esrrb*, *Nanog*, *Oct4*, and *Sall4*, were all substantially elevated (Additional file 2: Figure S4), affirming the association between alternative splicing of these isoforms and the maintenance of pluripotency.

These observations suggest a collaborative regulatory role of hnRNPK and SOX2 in shaping pluripotency through splicing modulation. For validation, we examined their collaborative role in splicing using the *Eif4a2* minigene reporter containing the alternative exon (Exon 11) of *Eif4a2*^*PTC*^ transcript in E14 ESCs (Fig. 4F). We noted a significant decrease of Exon 11 inclusion in ESCs following the individual KD of *hnRNPK* or *Sox2* (Fig. 4G). Notably, this reduction was more pronounced when *hnRNPK* and *Sox2* were simultaneously knocked down (Fig. 4G). In contrast, the splicing of *Gapdh* remained unaffected by the KD of *hnRNPK* or/and *Sox2* (Fig. 4G). These results align with our initial observation that this specific *Eif4a2* exon serves as a common splicing target for both hnRNPK and SOX2. In sum, these findings provide the evidence of a collaborative splicing role between hnRNPK and SOX2.

The RBM domain of SOX2 exhibits a preference for G/C-rich RNA sequences, potentially contributing to exon selection by interacting with the 5’ splice site during reprogramming [9]. On the other hand, hnRNPK binds to C(U)-rich elements and regulates pre-mRNA splicing at the 3’ splicing site [45]. When comparing the sequences around the splicing sites for exons affected by KD of *Sox2* or *hnRNPK*, we observed similar G/C-rich sequences around the 5’ splice site and CU-rich sequences around the 3’ splice site for exons undergoing alternative splicing in both groups (Additional file 2: Figure S5). In contrast, the sequences around the splicing sites for exons affected by KD of *Cdk9* are relatively different from those affected by KD of *Sox2* or *hnRNPK* (Additional file 2: Figure S5). Consequently, we hypothesized the potential involvement of hnRNPK in the interaction between SOX2 and the splicing targets. To test this, we performed RIP using E14 ESCs expressing GST-SOX2 and found that *hnRNPK* KD led to an approximately twofold decrease in the binding of SOX2 to the *Eif4a2* transcript (Fig. 4H). As an additional control, we performed RIP employing E14 ESCs expressing GST-SOX2-ΔRBM, which is a construct of SOX2 lacking the RNA-binding domain with a preference for G/C-rich sequences. Our RIP data indicated that the reduction of hnRNPK has no discernible effect either on the binding of SOX2-ΔRBM to the *Eif4a2* transcript (Fig. 4I) or on the splicing of the *Eif4a2* transcript in the minigene system (Fig. 4J). Altogether, these results suggest that hnRNPK collaborates with SOX2 in splicing by augmenting the binding of SOX2 to its target RNAs.

## Discussion

Transcription factors are central to determining cell fate by harmonizing gene expression across various molecular levels. They fine-tune gene expression by orchestrating RNA fate at both the transcriptional and splicing stages. The involvement of transcription factors in either transcription or alternative splicing depends on the specific partners with which they interact [8, 9, 46]. SOX2, a crucial determinant of cell fate, governs the balance between stemness and differentiation in ESCs by influencing transcription and splicing programs [8, 9, 47]. In the interactome of SOX2, alongside an enrichment of transcriptional regulators, there is a subset of interactors have versatile functions, especially a group of hnRNPs [13, 14, 48]. Nevertheless, the specific co-functions of these interactors remain largely unexplored. Recent studies have begun to shed light on the roles of hnRNPA2/B1 and hnRNPK. It has been demonstrated that the nuclear lncRNA RMST interacts with hnRNPA2/B1 and SOX2, collectively regulating the expression of SOX2 target genes [49]. hnRNPK is linked to various cellular processes, including transcription activation, pre-mRNA splicing, mRNA stability control, and the regulation of mRNA translation [36]. Numerous studies have underscored the significance of hnRNPK in pluripotent stem cells and development. However, its role in ESCs remains elusive. In one study, *hnRNPK* KD led to the downregulation of OCT4 and NANOG in murine ESCs, resulting in reduced cellular proliferation [50]. Conversely, an RNA-seq analysis provided different insights, indicating that *hnRNPK* KD has no effect on the expression of *Oct4* or *Nanog* and does not lead to overt differentiation [51]. A recent research revealed that in ESCs, hnRNPK co-localizes with SOX2 and OCT4 at pluripotency-related gene sites on chromatin but plays a minimal role in the transcriptional regulation of *Oct4* [22]. This observation implies that hnRNPK might not primarily function as a transcriptional regulator for pluripotency-related genes alongside SOX2 in ESCs.

In our study, we first investigated the roles of hnRNPK and SOX2 in transcriptional regulation for the maintenance of pluripotency in mESCs. During ESC differentiation, both hnRNPK and SOX2 gradually disengage from the chromatin (Fig. 1A, B). KD of either *hnRNPK* or *Sox2* prompts ESC differentiation (Fig. 1C, G). It’s noteworthy that their impact on pluripotency appears to be mutually dependent. KD of *hnRNPK* results in a decrease in *Sox2* expression, and vice versa (Additional file 2: Figure S1). Similar to OCT4, hnRNPK directly associates with SOX2 at its HMG domain (Fig. 2A-C, E) [4], suggesting hnRNPK’s potential involvement in transcription modulated by Sox2. As a transcription regulator, hnRNPK enhances the binding strength of CTCF at chromatin boundaries, thereby promoting the transcription of developmental genes [52]. Distinctively, the interaction between hnRNPK and the HMG domain of SOX2 doesn't necessarily imply a direct influence on DNA binding. Our findings indicate that hnRNPK does not influence the transcription induced by SOX2 through enhanced DNA binding (Fig. 3A-C). In mESCs, hnRNPK has been found to bind to the enhancer region of the *Oct4* gene along with SOX2 and other factors. However, reducing hnRNPK expression by approximately 50% did not significantly affect the mRNA levels of *Oct4*, indicating that hnRNPK may not directly modulate the binding of SOX2 to DNA. Notably, hnRNPK predominantly targets open chromatin regions in ESCs [22]. Therefore, its interaction with the HMG domain of SOX2 may primarily facilitate chromatin accessibility rather than directly influencing DNA binding. Nevertheless, hnRNPK's role appears to extend beyond this, as the abundance of the hnRNPK-SOX2 complex significantly affects the maintenance of ESC pluripotency (Figs. 1C-G, 2D, F, G).

P-TEFb governs the transcription elongation phase by Pol II, countering negative elongation factors and enhancing transcript elongation and processing [53]. In dividing cells, P-TEFb activity is regulated negatively when it binds with HEXIM1/2 in the 7SK snRNP complex and positively by various transcription factors and the super elongation complex [53]. Beyond its role in the transcription initiation, hnRNPK also plays a necessary role in the partitioning of 7SK snRNA among distinct ribonucleoproteins (RNPs) [54]. Additionally, SOX2 directly interacts with the P-TEFb subunit CYCLINT1 (Fig. 3D, F). We hypothesized that hnRNPK may modulate Pol II-mediated transcription elongation of SOX2 targeted genes. Interestingly, akin to its regulation of *Oct4* [22], hnRNPK does not significantly impact on the transcriptional regulation of SOX2. Despite the interaction of SOX2 and hnRNPK in the P-TEFb-interactome (Fig. 3D-F) and hnRNPK’s ability to rescue 7SK snRNA from Hexim1 (Fig. 3G-I, the RNA-seq data reveals that the expression of only a few genes is perturbed simultaneously in *hnRNPK*-KD, *Sox2*-KD and *Cdk9*-KD ESCs (Fig. 3J). Nevertheless, being part of the P-TEFb interactome signifies more than mere participation in transcription elongation, as P-TEFb also serves as a platform for alternative splicing of nascent pre-mRNA [55]. Though P-TEFb does not cooperate with SOX2 and hnRNPK in transcription, it may still serve as a platform for them in alternative splicing.

Both hnRNPK and SOX2 are reported as splicing regulators with roles in the regulation of alternative splicing events in cells [9, 19]. Despite their divergent effects on transcriptional regulation, hnRNPK and SOX2 consistently exert influence on pluripotency at the splicing level in ESCs (Additional file 2: Figure S2, Additional file 2: Figure S3). This phenomenon of disparate effects on transcriptional and splicing targets has been observed in the interaction between SOX9, another member of SOX transcription factors, and Y14, a component of the core exon junction complex, where Y14 shares 60% of SOX9 splicing targets but less than 5% of its transcriptional targets [56]. Moreover, analysis of a transcriptome dataset from the mouse fetal and adult cerebral cortex revealed nearly 400 differential alternative splicing events, with 31% of the genes found to be differentially regulated by alternative splicing during brain development showing no change in their total expression levels [57]. Upon comparing RNA-seq data from *hnRNPK*-KD and *Sox2*-KD ESCs, we have identified 819 alternative splicing events in 301 genes that changed in both types of ESCs, with 82.7% (677) of splicing events occurring in the same direction (Fig. 4A). Analysis of ΔPSI for these events revealed a strong correlation (R = 0.89, P-value < 2.2e-16) between splicing events after the KD of *hnRNPK* and those following the KD of *Sox2* (Additional file 2: Figure S3A), suggesting potential co-regulation of splicing by hnRNPK and SOX2. Among these genes with altered alternative splicing, some are pivotal for maintaining pluripotency (Additional file 1: Table S2), including transcription factors (ASH2L, SON, MAX, MTF2, SRSF11) [40, 58–61], splicing regulators (DDX56, MBNL1, hnRNPF) [62–64], and a translation initiation factor (eIF4A2) [43]. SOX2 has been suggested to play a role in co-transcriptionally regulating RNA metabolism [9]. We validated the alternative splicing of the transcription factor *Ash2l* and the translation initiation factor *Eif4a2*, both of which have a proximal SOX2 binding in mESCs [65] and are among the top 10 genes with the highest occurrence of alternative splicing events out of 677 splicing events (Additional file 2: Figure S3B), using RT-PCR. *Ash2l-b* and *Eif4a2*^*PTC*^ are key splicing isoforms essential for maintaining pluripotency in ESCs (Fig. 4E, Additional file 2: Figure S4) [41, 43]. Compared to the control group of ESCs, the reduction of either SOX2 or hnRNPK resulted in decreased levels of *Ash2l-b* and *Eif4a2*^*PTC*^, and a simultaneous KD of both *Sox2* and *hnRNPK* further intensified the reduction (Fig. 4B-D, F-G). Therefore, the maintenance of ESC pluripotency is likely orchestrated through the collaborative regulation of the splicing process by hnRNPK and SOX2 in a co-transcriptional manner, and the RBM domain of SOX2, operating independently of the HMG DNA-binding domain, is plausibly the functional component collaborating with hnRNPK.

hnRNPs contain diverse RNA-binding domains, including RRM and KH domains [17]. Functioning as alternative splicing regulators, they can either activate or repress specific splice sites based on the location of their binding site and interactions with other factors [66]. hnRNPK exhibits a strong and selective binding affinity to transcripts or promoters, primarily at poly(C) or CU-rich RNA stretches [45, 67]. SOX2 is implicated in splicing processes due to its RNA-binding activity. It exhibits a high affinity for G/C-rich RNA, particularly RNA with a poly(C) core [9]. We identified comparable G/C-rich sequences adjacent to the 5’ splice site and CU-rich sequences adjacent to the 3’ splice site in exons undergoing alternative splicing in both *hnRNPK*-KD and *Sox2*-KD E14 ESCs (Additional file 2: Figure S5). This finding suggests the intriguing possibility that hnRNPK may modulate splicing by collaborating with SOX2 in the binding to pre-mRNA targets. As expected, KD of *hnRNPK* reduces the binding of SOX2 to its target RNA (*Eif4a2*) and consequently influences alternative splicing (Fig. 4H). Both the HMG domain and RBM domain of SOX2 exhibit RNA-binding activity, but only the RBM domain possesses a G/C preference. Therefore, even lacking the RBM domain, SOX2-ΔRBM can still bind the *Eif4a2* transcript, but with a decrease (Fig. 4I). However, without the RBM domain, the KD of *hnRNPK* does not affect either its binding to the *Eif4a2* transcript or the alternative splicing pattern of the *Eif4a2* transcript (Fig. 4H-J), suggesting that the coordination of SOX2 with hnRNPK in splicing is determined by its RBM domain. This result confirms that SOX2’s role in splicing regulation is distinct from its HMG domain-related functions [9]. Take together, our data suggest that hnRNPK collaborates with SOX2 in splicing by enhancing the binding of Sox2 to its target RNAs.

Furthermore, SOX2 is involved in the transcription elongation machinery (Fig. 3D, F). This observation implies that SOX2 links transcription, splicing and transcription elongation. The intricate interplay between the elongation machinery and transcription factors forms a critical regulatory layer in both transcription and splicing processes. For instance, c-MYC engages in an interaction with the elongation factor SPT5, recruiting it to promoters and thereby augmenting the processivity of Pol II [68]. Moreover, c-MYC binds to the promoter of the RNA-binding protein *Sam68*, exerting regulatory control over both its expression and splicing dynamics. Specifically, c-MYC modulates *Sam68* splicing by influencing the variation in Pol II elongation rates [69]. Future research endeavors should delve into unraveling the intricate crosstalk between SOX2-regulated transcription and splicing, particularly in association with the transcription elongation machinery.

## Conclusions

**Fig. 5:**
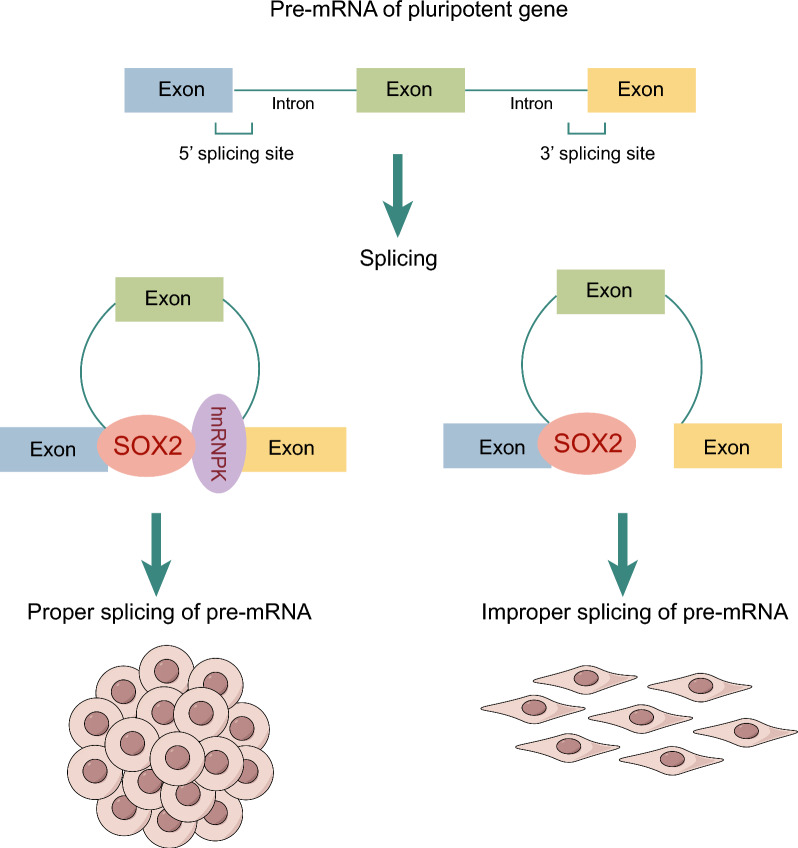
Proposed mechanism for the coordinated regulation of splicing by SOX2 and hnRNPK. hnRNPK boosts SOX2 interaction with target pre-mRNAs of pluripotent genes and collaborates in regulating alternative splicing to maintain ESC pluripotency

In summary, our investigation unveils a previously unrecognized collaborative role for SOX2 and hnRNPK in the regulation of alternative splicing during pluripotency maintenance (Fig. 5). SOX2 directly interacts with hnRNPK via its HMG domain, and they colocalize in the chromatin, playing essential roles in maintaining ESC pluripotency. Despite both hnRNPK and SOX2 participating in transcription and being associated with the transcription elongation machinery, hnRNPK has a limited impact on the transcriptional regulation of SOX2. The modulation of splicing by hnRNPK involves the enhancement of SOX2 binding to its target pre-mRNAs. These data mark the inception of the SOX2-mediated alternative splicing network.

### Supplementary Information

Additional file 1.

Additional file 2.

## Abbreviations

hnRNP: Heterogeneous nuclear ribonucleoprotein
HMG: High-mobility group
RBM: RNA binding motif
E14 ESC: E14Tg2A embryonic stem cells
ESC: Embryonic stem cells
LIF: Leukemia inhibitory factor
qRT-PCR: Quantitative real-time PCR
ALP: Alkaline phosphatase
co-IP: Co-immunoprecipitation
IF: Immunofluorescence staining
EMSA: Electrophoretic mobility shift assay
RIP: RNA immunoprecipitation assay
RNA-seq: RNA-sequencing
PWM: Position weight matrix
RA: Retinoic acid
KD: Knockdown
WT: Wild-type
MEF: Mouse embryonic fibroblasts
GST: Glutathione-S-transferase
RNA polymerase II: Pol II
snRNP: Small nuclear ribonucleoprotein

## References

[1] Spitz F, Furlong EE. Transcription factors: from enhancer binding to developmental control. Nat Rev Genet. 2012;13(9):613–26. 22868264 10.1038/nrg3207

[2] Vierbuchen T, Wernig M. Direct lineage conversions: unnatural but useful? Nat Biotechnol. 2011;29(10):892–907. 21997635 10.1038/nbt.1946 PMC3222779

[3] Sato T, et al. Epigenomic profiling discovers trans-lineage SOX2 partnerships driving tumor heterogeneity in lung squamous cell carcinoma. Cancer Res. 2019;79(24):6084–100. 31551362 10.1158/0008-5472.CAN-19-2132 PMC6911633

[4] Michael AK, et al. Mechanisms of OCT4-SOX2 motif readout on nucleosomes. Science. 2020;368(6498):1460–5. 32327602 10.1126/science.abb0074

[5] Soufi A, et al. Pioneer transcription factors target partial DNA motifs on nucleosomes to initiate reprogramming. Cell. 2015;161(3):555–68. 25892221 10.1016/j.cell.2015.03.017 PMC4409934

[6] Michael AK, Thomä NH. Reading the chromatinized genome. Cell. 2021;184(14):3599–611. 34146479 10.1016/j.cell.2021.05.029

[7] Zhang S, et al. OCT4 and PAX6 determine the dual function of SOX2 in human ESCs as a key pluripotent or neural factor. Stem Cell Res Ther. 2019;10(1):122. 30999923 10.1186/s13287-019-1228-7 PMC6471829

[8] Zhang Y, Hou L. Alternate roles of sox transcription factors beyond transcription initiation. Int J Mol Sci. 2021;22(11):5949. 34073089 10.3390/ijms22115949 PMC8198692

[9] Hou L, et al. Concurrent binding to DNA and RNA facilitates the pluripotency reprogramming activity of Sox2. Nucleic Acids Res. 2020;48(7):3869–87. 32016422 10.1093/nar/gkaa067 PMC7144947

[10] Jing R, et al. Long noncoding RNA Q associates with Sox2 and is involved in the maintenance of pluripotency in mouse embryonic stem cells. Stem Cells. 2020;38(7):834–48. 32277787 10.1002/stem.3180

[11] Holmes ZE, et al. The Sox2 transcription factor binds RNA. Nat Commun. 2020;11(1):1805. 32286318 10.1038/s41467-020-15571-8 PMC7156710

[12] Han H, et al. Multilayered control of alternative splicing regulatory networks by transcription factors. Mol Cell. 2017;65(3):539-553.e7. 28157508 10.1016/j.molcel.2017.01.011

[13] Fang X, et al. Landscape of the SOX2 protein-protein interactome. Proteomics. 2011;11(5):921–34. 21280222 10.1002/pmic.201000419

[14] Mallanna SK, et al. Proteomic analysis of Sox2-associated proteins during early stages of mouse embryonic stem cell differentiation identifies Sox21 as a novel regulator of stem cell fate. Stem Cells. 2010;28(10):1715–27. 20687156 10.1002/stem.494 PMC3260005

[15] Saud K, et al. SFPQ associates to LSD1 and regulates the migration of newborn pyramidal neurons in the developing cerebral cortex. Int J Dev Neurosci. 2017;57:1–11. 28034769 10.1016/j.ijdevneu.2016.12.006

[16] Samudyata, et al. Interaction of Sox2 with RNA binding proteins in mouse embryonic stem cells. Exp Cell Res. 2019;381(1):129–38. 31077711 10.1016/j.yexcr.2019.05.006 PMC6994247

[17] Martinez-Contreras R, et al. hnRNP proteins and splicing control. Adv Exp Med Biol. 2007;623:123–47. 18380344 10.1007/978-0-387-77374-2_8

[18] Geuens T, Bouhy D, Timmerman V. The hnRNP family: insights into their role in health and disease. Hum Genet. 2016;135(8):851–67. 27215579 10.1007/s00439-016-1683-5 PMC4947485

[19] Aitken MJL, et al. Heterogeneous nuclear ribonucleoprotein K is overexpressed in acute myeloid leukemia and causes myeloproliferation in mice via altered Runx1 splicing. NAR Cancer. 2022. 10.1093/narcan/zcac039. 36518526 10.1093/narcan/zcac039 PMC9732523

[20] Kong X, et al. LncRNA-Smad7 mediates cross-talk between Nodal/TGF-β and BMP signaling to regulate cell fate determination of pluripotent and multipotent cells. Nucleic Acids Res. 2022;50(18):10526–43. 36134711 10.1093/nar/gkac780 PMC9561265

[21] Li J, et al. HNRNPK maintains epidermal progenitor function through transcription of proliferation genes and degrading differentiation promoting mRNAs. Nat Commun. 2019;10(1):4198. 31519929 10.1038/s41467-019-12238-x PMC6744489

[22] Bakhmet EI, et al. hnRNP-K targets open chromatin in mouse embryonic stem cells in concert with multiple regulators. Stem Cells. 2019;37(8):1018–29. 31021473 10.1002/stem.3025

[23] Sigova AA, et al. Transcription factor trapping by RNA in gene regulatory elements. Science. 2015;350(6263):978–81. 26516199 10.1126/science.aad3346 PMC4720525

[24] Llano M, et al. Identification and characterization of the chromatin-binding domains of the HIV-1 integrase interactor LEDGF/p75. J Mol Biol. 2006;360(4):760–73. 16793062 10.1016/j.jmb.2006.04.073

[25] Heyd F, Lynch KW. Phosphorylation-dependent regulation of PSF by GSK3 controls CD45 alternative splicing. Mol Cell. 2010;40(1):126–37. 20932480 10.1016/j.molcel.2010.09.013 PMC2954053

[26] Lee TI, Johnstone SE, Young RA. Chromatin immunoprecipitation and microarray-based analysis of protein location. Nat Protoc. 2006;1(2):729–48. 17406303 10.1038/nprot.2006.98 PMC3004291

[27] Keene JD, Komisarow JM, Friedersdorf MB. RIP-Chip: the isolation and identification of mRNAs, microRNAs and protein components of ribonucleoprotein complexes from cell extracts. Nat Protoc. 2006;1(1):302–7. 17406249 10.1038/nprot.2006.47

[28] Bolger AM, Lohse M, Usadel B. Trimmomatic: a flexible trimmer for Illumina sequence data. Bioinformatics. 2014;30(15):2114–20. 24695404 10.1093/bioinformatics/btu170 PMC4103590

[29] Dobin A, et al. STAR: ultrafast universal RNA-seq aligner. Bioinformatics. 2013;29(1):15–21. 23104886 10.1093/bioinformatics/bts635 PMC3530905

[30] Robinson MD, McCarthy DJ, Smyth GK. edgeR: a bioconductor package for differential expression analysis of digital gene expression data. Bioinformatics. 2009;26(1):139–40. 19910308 10.1093/bioinformatics/btp616 PMC2796818

[31] Shen S, et al. rMATS: robust and flexible detection of differential alternative splicing from replicate RNA-Seq data. Proc Natl Acad Sci USA. 2014;111(51):E5593–601. 25480548 10.1073/pnas.1419161111 PMC4280593

[32] Vitting-Seerup K, Sandelin A. IsoformSwitchAnalyzeR: analysis of changes in genome-wide patterns of alternative splicing and its functional consequences. Bioinformatics. 2019;35(21):4469–71. 30989184 10.1093/bioinformatics/btz247

[33] Mikula M, et al. Landscape of the hnRNP K protein-protein interactome. Proteomics. 2006;6(8):2395–406. 16518874 10.1002/pmic.200500632

[34] Pintacuda G, et al. hnRNPK recruits PCGF3/5-PRC1 to the Xist RNA B-repeat to establish polycomb-mediated chromosomal silencing. Mol Cell. 2017;68(5):955-969.e10. 29220657 10.1016/j.molcel.2017.11.013 PMC5735038

[35] Leal G, et al. The RNA-binding protein hnRNP K mediates the effect of BDNF on dendritic mRNA metabolism and regulates synaptic NMDA receptors in hippocampal neurons. eNeuro. 2017. 10.1523/ENEURO.0268-17.2017. 29255796 10.1523/ENEURO.0268-17.2017 PMC5732018

[36] Bomsztyk K, Denisenko O, Ostrowski J. hnRNP K: one protein multiple processes. BioEssays. 2004;26(6):629–38. 15170860 10.1002/bies.20048

[37] Pham VV, et al. A structure-based mechanism for displacement of the HEXIM adapter from 7SK small nuclear RNA. Commun Biol. 2022;5(1):819. 35970937 10.1038/s42003-022-03734-w PMC9378691

[38] Ji C, et al. Interaction of 7SK with the Smn complex modulates snRNP production. Nat Commun. 2021;12(1):1278. 33627647 10.1038/s41467-021-21529-1 PMC7904863

[39] Muniz L, et al. Controlling cellular P-TEFb activity by the HIV-1 transcriptional transactivator tat. PLoS Pathog. 2010;6(10): e1001152. 20976203 10.1371/journal.ppat.1001152 PMC2954905

[40] Tsai PH, et al. Ash2l interacts with Oct4-stemness circuitry to promote super-enhancer-driven pluripotency network. Nucleic Acids Res. 2019;47(19):10115–33. 31555818 10.1093/nar/gkz801 PMC6821267

[41] Li S, et al. Disruption of OCT4 ubiquitination increases OCT4 protein stability and ASH2L-B-mediated H3K4 methylation promoting pluripotency acquisition. Stem Cell Rep. 2018;11(4):973–87. 10.1016/j.stemcr.2018.09.001 PMC6178847 30269953

[42] Li D, et al. eIF4A2 targets developmental potency and histone H3.3 transcripts for translational control of stem cell pluripotency. Sci Adv. 2022;8(13):eabm0478. 35353581 10.1126/sciadv.abm0478 PMC8967233

[43] Huth M, et al. NMD is required for timely cell fate transitions by fine-tuning gene expression and regulating translation. Genes Dev. 2022;36(5–6):348–67. 35241478 10.1101/gad.347690.120 PMC8973849

[44] Murata K, et al. PRMT1 deficiency in mouse juvenile heart induces dilated cardiomyopathy and reveals cryptic alternative splicing products. iScience. 2018;8:200–13. 30321814 10.1016/j.isci.2018.09.023 PMC6197527

[45] Thisted T, Lyakhov DL, Liebhaber SA. Optimized RNA targets of two closely related triple KH domain proteins, heterogeneous nuclear ribonucleoprotein K and alphaCP-2KL, suggest Distinct modes of RNA recognition. J Biol Chem. 2001;276(20):17484–96. 11278705 10.1074/jbc.M010594200

[46] Salomonis N, et al. Alternative splicing regulates mouse embryonic stem cell pluripotency and differentiation. Proc Natl Acad Sci USA. 2010;107(23):10514–9. 20498046 10.1073/pnas.0912260107 PMC2890851

[47] Xiong L, et al. Oct4 differentially regulates chromatin opening and enhancer transcription in pluripotent stem cells. eLife. 2022;11:e71533. 35621159 10.7554/eLife.71533 PMC9142147

[48] Samudyata, et al. Interaction of Sox2 with RNA binding proteins in mouse embryonic stem cells. Exp Cell Res. 2019;381(1):129–38. 31077711 10.1016/j.yexcr.2019.05.006 PMC6994247

[49] Ng S-Y, et al. The Long Noncoding RNA RMST Interacts with SOX2 to Regulate Neurogenesis. Mol Cell. 2013;51(3):349–59. 23932716 10.1016/j.molcel.2013.07.017

[50] Lin N, et al. An evolutionarily conserved long noncoding RNA TUNA controls pluripotency and neural lineage commitment. Mol Cell. 2014;53(6):1005–19. 24530304 10.1016/j.molcel.2014.01.021 PMC4010157

[51] Thompson PJ, et al. hnRNP K coordinates transcriptional silencing by SETDB1 in embryonic stem cells. PLoS Genet. 2015;11(1): e1004933. 25611934 10.1371/journal.pgen.1004933 PMC4303303

[52] Chen Y, et al. Hnrnpk is essential for embryonic limb bud development as a transcription activator and a collaborator of insulator protein Ctcf. Cell Death Differ. 2023;30(10):2293–308. 37608075 10.1038/s41418-023-01207-z PMC10589297

[53] Fujinaga K, Huang F, Peterlin BM. P-TEFb: The master regulator of transcription elongation. Mol Cell. 2023;83(3):393–403. 36599353 10.1016/j.molcel.2022.12.006 PMC9898187

[54] Hogg JR, Collins K. RNA-based affinity purification reveals 7SK RNPs with distinct composition and regulation. RNA. 2007;13(6):868–80. 17456562 10.1261/rna.565207 PMC1869041

[55] Lenasi T, Barboric M. P-TEFb stimulates transcription elongation and pre-mRNA splicing through multilateral mechanisms. RNA Biol. 2010;7(2):145–50. 20305375 10.4161/rna.7.2.11057

[56] Girardot M, et al. SOX9 has distinct regulatory roles in alternative splicing and transcription. Nucleic Acids Res. 2018;46(17):9106–18. 29901772 10.1093/nar/gky553 PMC6158501

[57] Dillman AA, et al. mRNA expression, splicing and editing in the embryonic and adult mouse cerebral cortex. Nat Neurosci. 2013;16(4):499–506. 23416452 10.1038/nn.3332 PMC3609882

[58] Lu X, et al. SON connects the splicing-regulatory network with pluripotency in human embryonic stem cells. Nat Cell Biol. 2013;15(10):1141–52. 24013217 10.1038/ncb2839 PMC4097007

[59] Chappell J, et al. MYC/MAX control ERK signaling and pluripotency by regulation of dual-specificity phosphatases 2 and 7. Genes Dev. 2013;27(7):725–33. 23592794 10.1101/gad.211300.112 PMC3639414

[60] Zhang Z, et al. PRC2 complexes with JARID2, MTF2, and esPRC2p48 in ES cells to modulate ES cell pluripotency and somatic cell reprogramming. Stem Cells. 2011;29(2):229–40. 21732481 10.1002/stem.578 PMC3711030

[61] Kanitz A, et al. Conserved regulation of RNA processing in somatic cell reprogramming. BMC Genomics. 2019;20(1):100. 30704403 10.1186/s12864-019-5438-2 PMC6357513

[62] Wang J, et al. Ddx56 maintains proliferation of mouse embryonic stem cells via ribosome assembly and interaction with the Oct4/Sox2 complex. Stem Cell Res Ther. 2020;11(1):314. 32703285 10.1186/s13287-020-01800-w PMC7376950

[63] Venables JP, et al. MBNL1 and RBFOX2 cooperate to establish a splicing programme involved in pluripotent stem cell differentiation. Nat Commun. 2013;4:2480. 24048253 10.1038/ncomms3480

[64] Yamazaki T, et al. TCF3 alternative splicing controlled by hnRNP H/F regulates E-cadherin expression and hESC pluripotency. Genes Dev. 2018;32(17–18):1161–74. 30115631 10.1101/gad.316984.118 PMC6120717

[65] Kim KY, et al. Uhrf1 regulates active transcriptional marks at bivalent domains in pluripotent stem cells through Setd1a. Nat Commun. 2018;9(1):2583. 29968706 10.1038/s41467-018-04818-0 PMC6030064

[66] Xue Y, et al. Genome-wide analysis of PTB-RNA interactions reveals a strategy used by the general splicing repressor to modulate exon inclusion or skipping. Mol Cell. 2009;36(6):996–1006. 20064465 10.1016/j.molcel.2009.12.003 PMC2807993

[67] Makeyev AV, Liebhaber SA. The poly(C)-binding proteins: a multiplicity of functions and a search for mechanisms. RNA. 2002;8(3):265–78. 12003487 10.1017/S1355838202024627 PMC1370249

[68] Baluapuri A, et al. MYC recruits SPT5 to RNA polymerase II to promote processive transcription elongation. Mol Cell. 2019;74(4):674-687.e11. 30928206 10.1016/j.molcel.2019.02.031 PMC6527870

[69] Caggiano C, et al. c-MYC empowers transcription and productive splicing of the oncogenic splicing factor Sam68 in cancer. Nucleic Acids Res. 2019;47(12):6160–71. 31066450 10.1093/nar/gkz344 PMC6614821

