# Supplementary material for: SOX2 interacts with hnRNPK to modulate alternative splicing in mouse embryonic stem cells
Source: Cell Biosci. 2024 Aug 19;14:102. doi: 10.1186/s13578-024-01284-8 (PMC11331657; doi:10.1186/s13578-024-01284-8)
Supplement: Supplementary file 1 — Additional file 1. [file 13578_2024_1284_MOESM1_ESM.zip › Supplementary material/Additional file 1 Table S1.docx]

**Table S1.** Oligos used in this paper

|  | Sequence (5'-3') |
| --- | --- |
| **Oligonucleotides sequence of shRNA** | |
| NG-shRNA-forward | CCGGTTCTCCGAACGTGTCACGTCTCGAGACGTGACACGTTCGGAGAATTTTTG |
| NG-shRNA-reverse | AATTCAAAAATTCTCCGAACGTGTCACGTCTCGAGACGTGACACGTTCGGAGAA |
| SOX2-shRNA1-forward | CCGGTAGGAGCACCCGGATTATAAATCTCGAGATTTATAATCCGGGTGCTCCTTTTTTG |
| SOX2-shRNA1-reverse | AATTCAAAAAAGGAGCACCCGGATTATAAATCTCGAGATTTATAATCCGGGTGCTCCTA |
| SOX2-shRNA2-forward | CCGGTCAAAGAGATACAAGGGAATTGCTCGAGCAATTCCCTTGTATCTCTTTGTTTTTG |
| SOX2-shRNA2-reverse | AATTCAAAAACAAAGAGATACAAGGGAATTGCTCGAGCAATTCCCTTGTATCTCTTTGA |
| hnRNPK-shRNA1-forward | CCGGTCGGATTAAACAAATTCGTCATCTCGAGATGACGAATTTGTTTAATCCGTTTTTG |
| hnRNPK-shRNA1-reverse | AATTCAAAAACGGATTAAACAAATTCGTCATCTCGAGATGACGAATTTGTTTAATCCGA |
| hnRNPK-shRNA2-forward | CCGGTGGGTTCAGTGCTGATGAAACTCTCGAGAGTTTCATCAGCACTGAACCCTTTTTG |
| hnRNPK-shRNA2-reverse | AATTCAAAAAGGGTTCAGTGCTGATGAAACTCTCGAGAGTTTCATCAGCACTGAACCCA |
| CDK9-shRNA1-forward | CCGGTGGACATGAAGGCTGCGAATGTCTCGAGACATTCGCAGCCTTCATGTCCTTTTTG |
| CDK9-shRNA1-reverse | AATTCAAAAAGGACATGAAGGCTGCGAATGTCTCGAGACATTCGCAGCCTTCATGTCCA |
| CDK9-shRNA2-forward | CCGGTGCATGCTGTCTACTCACTTGACTCGAGTCAAGTGAGTAGACAGCATGCTTTTTG |
| CDK9-shRNA2-reverse | AATTCAAAAAGCATGCTGTCTACTCACTTGACTCGAGTCAAGTGAGTAGACAGCATGCA |
| **Primers used for splicing variant analysis** | |
| *Eif4a2*-AS-forward | GGCCCGTGGGATTGACG |
| *Eif4a2*-AS-reverse | CCTTTCCTCCCAAATCGACCCC |
| *Ash2l*-AS-forward | GGATGCAAACTTGGTTGATGTAAGTGG |
| *Ash2l*-AS-reverse | GGATGTTCATCCTGTGTTCGAGACTG |
| **Primers used for plasmid construction in the rescue experiment** | |
| *Eif4a2^PTC^*-forward | TTATACTTGGATCACCCATATGATGTCTGGTGGCTCCGCG |
| *Eif4a2^PTC^*-reverse | GCCTCCCCTACCCGGTAGAATTCCTATCGACTCCTGTGAATATAGTTTTCACGA |
| *Ash2l*-*b*-forward | \| TTATACTTGGATCACCCATATGATGGATACCCAGGCGGGC \| \| --- \| |
| *Ash2l*-*b*-reverse | GCCTCCCCTACCCGGTAGAATTCTTAGGGTTCCCAGGGTGGACT |
| **Primers used for protein expression plasmid construction** | |
| SOX2-BamHI-forward | CGGGATCCATGTATAACATGATGGAGACGGAGCTG |
| SOX2-XhoI-reverse | CCGCTCGAGTCACATGTGCGACAGGGGC |
| HMG-BamHI-forward | CGGGATCCGACCGCGTCAAGAGGCC |
| HMG-XhoI-reverse | CCGCTCGAGTCACTTGGTTTTCCGCCGCGG |
| SOX2-ΔHMG-forward | AACCAGAAGAACAGCCCGACGCTCATGAAGAAGGATAAGTACAC |
| SOX2-ΔHMG-reverse | ATCCTTCTTCATGAGCGTCGGGCTGTTCTTCTGGTTGC |
| hnRNPK-EcoRI-forward | CGGAATTCATGGAGACCGAACAGCCAGAAG |
| hnRNPK-XhoI-reverse | CCGCTCGAGTTAGAATCCTTCAACATCTGCATACTGCTTC |
| HEXIM1-forward | GTTCCGCGTGGATCCCCGGAATTCATGGCCGAGCCACTCTTGACA |
| HEXIM1-reverse | TCAGTCACGATGCGGCCGCTCGAGCTAGTCGCCGAACTTGGAAAGAGGC |
| SOX2-NcoI-forward | CATGCCATGGATGTATAACATGATGGAGACGGAGCT |
| SOX2-XhoI-reverse | TGCTCTAGATTAAACGGGCCCTCTAGACGC |
| CYCLIN T1-NcoI-forward | CATGCCATGGATGGAGGGAGAGAGGAAGAACAAC |
| CYCLIN T1-XhoI-reverse | CCGCTCGAGCTTGTCATCGTCATCCTTGTAATCGATG |
| SOX2-NheI-forward | CTAGCTAGCATGTATAACATGATGGAGACGGAGCTGAAG |
| SOX2-XbaI-reverse | TGCTCTAGACGCGTAATCTGGAACATCGTATGGGTA |
| hnRNPK-NheI-forward | CGGCTAGCTAGCATGGAGACCGAACAGCCAG |
| hnRNPK-XhoI-reverse | CCGCTCGAGTTAGAATCCTTCAACATCTGCATACTGCT |
| GST-SOX2-forward | CCTCGTAAAGGTCTAGAGCTAGCGTATGTCCCCTATACTAGGTTATTGGAAAA |
| GST-SOX2-reverse | CAGGGAGAAGTTAGTGGCCATGTGCGACAGGGG |
| GST-SOX2-ΔRBM-forward | ATGATGCAGGAGCAGCTGGGCTACCCGCAGCACC |
| GST-SOX2-ΔRBM-reverse | ATCCTTCTTCATGAGCGTCTTGGTTTTCCGCCGCG |
| His-hnRNPK-ΔKI-forward | TGTAAAACCACCATAATCATAGGTCTCATCATAAAAG |
| His-hnRNPK-ΔKI-reverse | TGTAAAACCACCATAATCATAGGTCTCATCATAAAAG |
| **Primers used for 7SK snRNA *in vitro* transcription** | |
| 7SK-SL1-forward | TAATACGACTCACTATAGGGATGTGAGGGCGATCT |
| 7SK-SL1-reverse | CGCACATGGAGCGGTGAGG |
| 7SK-snRNA-reverse | AAAAGAAAGGCAGACTGCCACATGC |
| **Primers used for dual luciferase assay and minigene constructions** | |
| *Fgf4* enhancer-KpnI-forward | GGGGTACCAGACTTCTGAGCAACCTCCCGAAT |
| *Fgf4* enhancer-NheI-reverse | CTAGCTAGCCAACTGTCTTCTCCCCAACACTCT |
| *Eif4a2*-NcoI-forward | CATGCCATGGGGCCCGTGGGATTGACG |
| *Eif4a2*-XbaI-reverse | GCTCTAGACCTTTCCTCCCAAATCGACCCC |
| **The underlined are restriction sites.** |  |


|  | Sequence (5'-3') |
| --- | --- |

| **Sequence of *Fgf4* enhancer** | |
| --- | --- |
| *Fgf4* enhancer | AGACTTCTGAGCAACCTCCCGAATTAACTTTATGGGAGGCTACAGACAGCAAGACTGGAAAATCTCATTGGCATTTTTTTTTTTTGTCTTTCACATTCCTTTAGAAAACTCTTTGTTTGGATGCTAATGGGATACTTAAAATACTATTCTGTACCACAGCCCAAGATGGAAGAAGCCACACCCCAAAGCTGAGGTGGGAGCTCCTCCCAAACTTCCTTTCTGTCTGGTGGCTCACAGGACAATAAGATTTTGTGTTTTTTAAATCCAGGCCCTAGGCTCCAAGAGTGTTGGGGAGAAGACAGTTG |
| **Sequences of 7SK snRNA constructs** | |
| 7SK snRNA | GGAUGUGAGGGCGAUCUGGCUGCGACAUCUGUCACCCCAUUGAUCGCCAGGGUUGAUUCGGCUGAUCUGGCUGGCUAGGCGGGUGUCCCCUUCCUCCCUCACCGCUCCAUGUGCGUCCCUCCCGAAGCUGCGCGCUCGGUCGAAGAGGACGACCUUCCCCGAAUAGAGGAGGACCGGUCUUCGGUCAAGGGUAUACGAGUAGCUGCGCUCCCCUGCUAGAACCUCCAAACAAGCUCUCAAGGUCCAUUGUAGGAGAACGUAGGGUAGUCAAGCUUCCAAGACUCCAGACACAUCCAAAUGAGGCGCUGCAUGUGGCAGUCUGCCUUUCUUUUUUUUUU |
| 7SK snRNA SL1 | GGATGTGAGGGCGATCTGGCTGCGACATCTGTCACCCCATTGATCGCCAGGGTTGATTCGGCTGATCTGGCTGGCTAGGCGGGTGTCCCCTTCCTCCCTCACCGCTCC |
