# Supplementary material for: SOX2 interacts with hnRNPK to modulate alternative splicing in mouse embryonic stem cells
Source: Cell Biosci. 2024 Aug 19;14:102. doi: 10.1186/s13578-024-01284-8 (PMC11331657; doi:10.1186/s13578-024-01284-8)

Additional file 2

Figure S1. Comparing the gene expression levels of *Sox2* and *hnRNPK* in mESCs. The relative transcript levels of endogenous *Sox2* and *hnRNPK* in control (shscramble), *Sox2*-KD (shSox2-1), and *hnRNPK*-KD (shRNPK-1) E14 ESCs were analyzed by qRT–PCR. Barplots represent the mean ± SD (n=3). ANOVA was used to assess statistical significance (*, **, and *** indicate P-values of <0.05, <0.01, and <0.001, respectively).

Figure S2. Relationship between differential isoform usage and differential gene expression. The X-axis represents the log2 fold change (log2FC) in gene expression between KD conditions (shhnRNPK, shSOX2) and the control (shscramble). The Y-axis represents the relative isoform fraction expression (dIF) in the same comparison. IF is quantified as the fraction of the parent gene originating from a specific isoform. Significant isoform usage events, defined by |dIF| > 0.1 and q-value < 0.05, are highlighted in red.

Figure S3. Correlation analysis of alternative splicing events affected by SOX2 and hnRNPK in the same direction. **A.** ΔPSI correlation revealed a strong correlation (R = 0.89, *P*-value < 2.2e-16) between alternative splicing (AS) events after the KD of *hnRNPK* and those following the KD of *Sox2*. The ΔPSI of *Eif4a2* and *Ash2l* AS events are indicated in red or green. Statistical significance was performed by Pearson correlation test. **B.** The top ten genes with the highest number of changed AS occurrence. The AS events used in (**A**) and (**B**) are from Figure 4A.

Figure S4. QRT-PCR assessment of the relative transcript levels of selected pluripotency markers in *hnRNPK*-KD (shhRNPK-2) and *Sox2*-KD (shSox2-2) E14 ESCs after 48 or 72 hours of Dox-induced expression of *Ash2l-b* and *Eif4a2*^{PTC}. Uninduced: before Dox induction. Data are represented as mean ± SD (n=3). ANOVA was used to assess significance (**** and ** indicate *P*-values of < 0.0001 and <0.01, respectively; ns indicates not significant).

Figure S5. Characterization of nucleotide composition around 5' splice sites and 3' splice sites. Nucleotide frequencies around the 5' splice sites and 3' splice sites of exons in alternative splicing effected by *Sox2* (**A**), *hnRNPK* (**B**) or *Cdk9* (**C**).

Figure S1

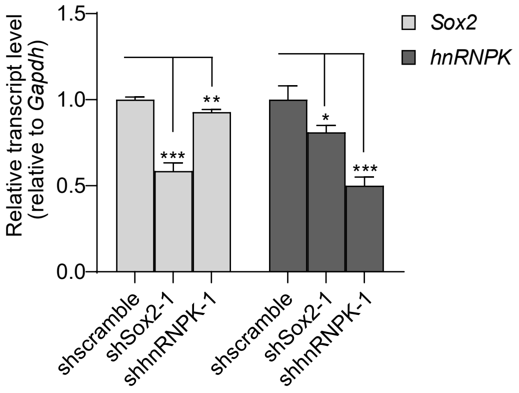

Relative transcript level (relative to Gapdh)
1.5
1.0
0.5
0.0
Sox2
hnRNPK
***
**
*
***
shscramble
shSox2-1
shhnRNPK-1
shscramble
shSox2-1
shhnRNPK-1

Figure S2

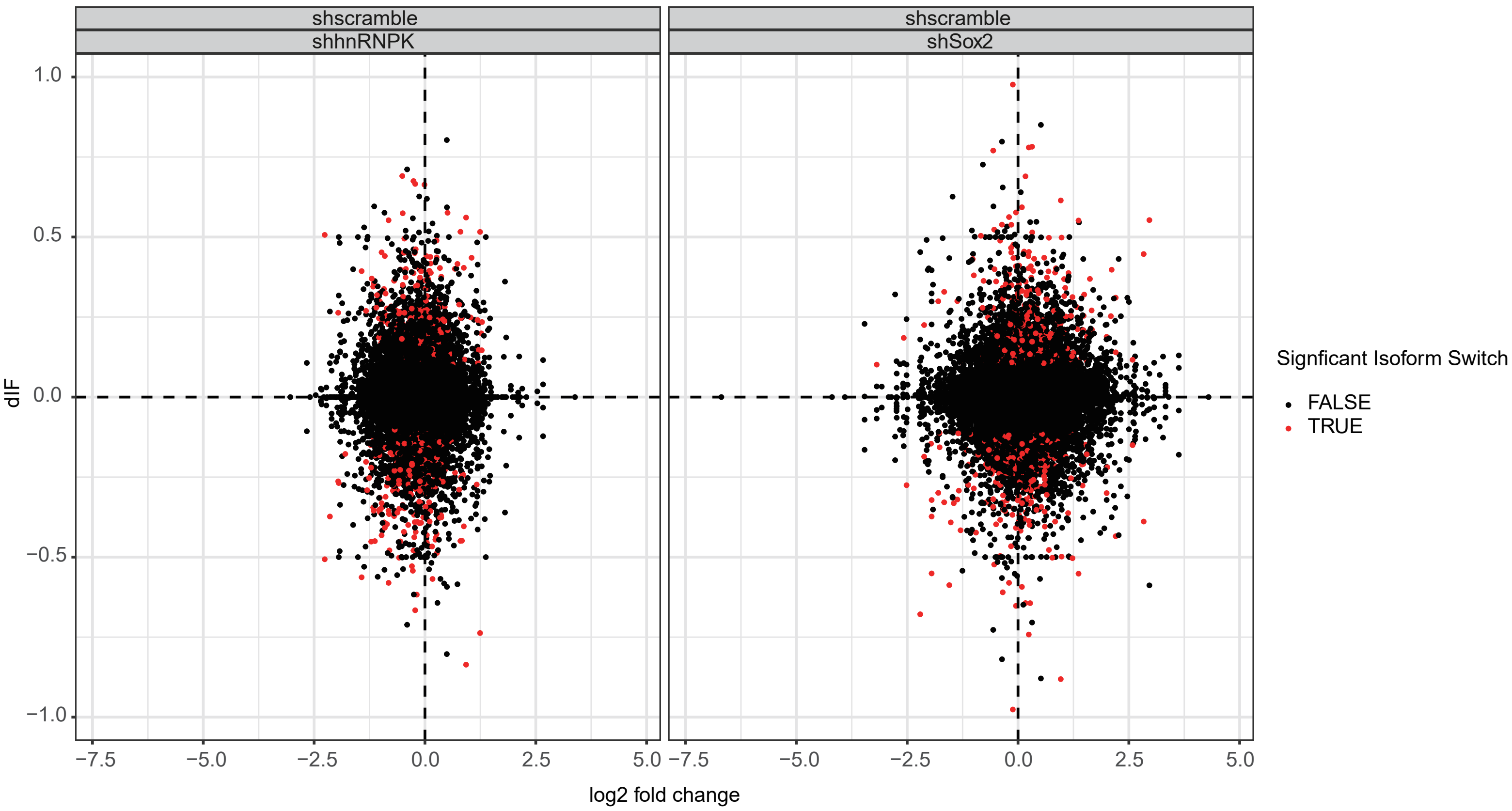

shscramble
shhnRNPK
shscramble
shSox2
1.0
0.5
0.0
−0.5
−1.0
dIF
−7.5
−5.0
−2.5
0.0
2.5
5.0
log2 fold change
Signficant Isoform Switch
FALSE
TRUE

Figure S3

A

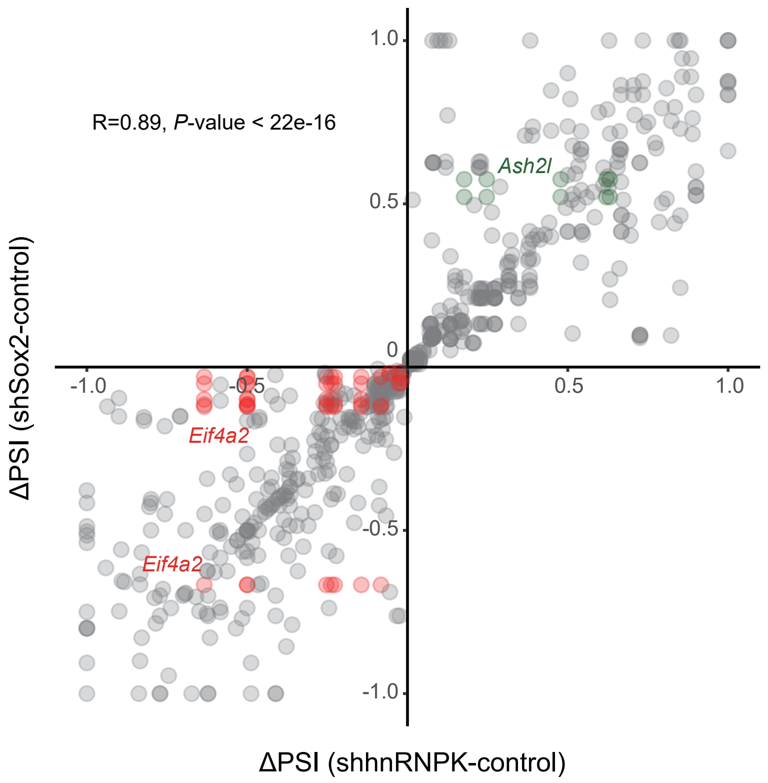
R=0.89, P-value < 22e-16
Ash2l
Eif4a2
Eif4a2
1.0
0.5
0
-0.5
-1.0
-1.0
-0.5
0.5
1.0
ΔPSI (shSox2-control)
ΔPSI (shhnRNPK-control)

B

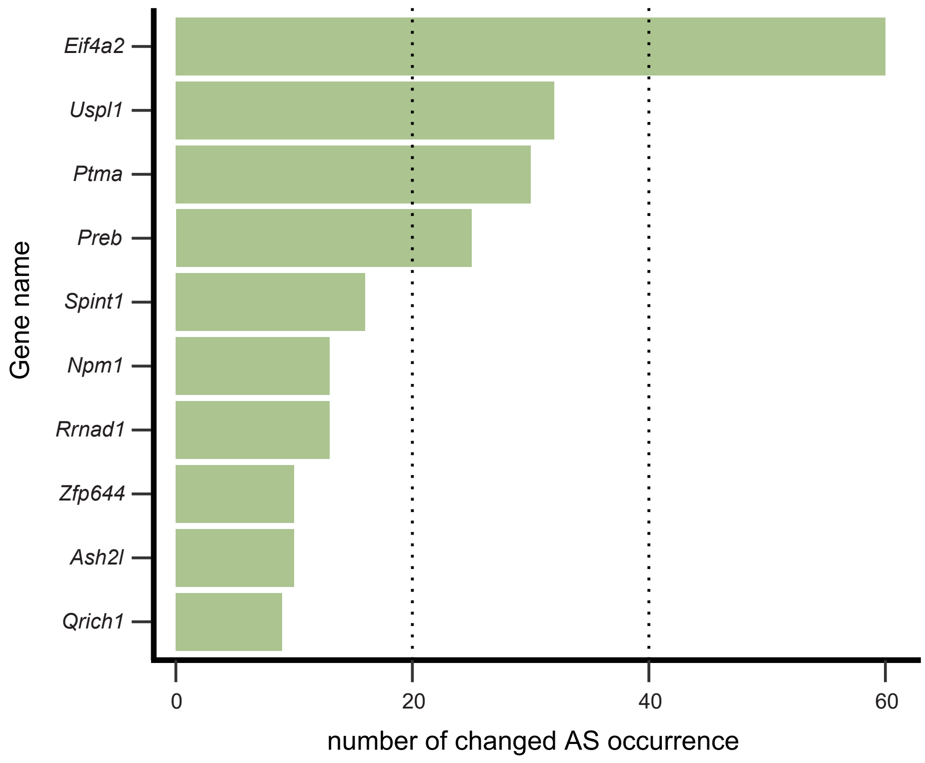
Eif4a2
Uspl1
Ptma
Preb
Spint1
Npm1
Rrnad1
Zfp644
Ash2l
Qrich1
Gene name
0
20
40
60
number of changed AS occurrence

Figure S4

A

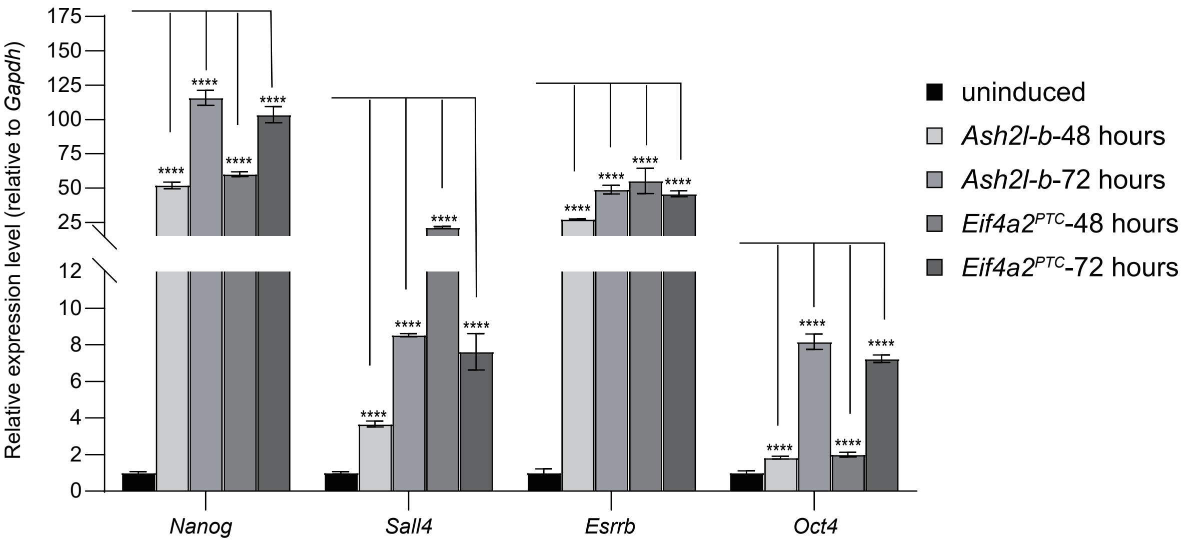

Relative expression level (relative to Gapdh)
175
150
125
100
75
50
25
12
10
8
6
4
2
0
Nanog
Sall4
Esrrb
Oct4
uninduced
Ash2l-b-48 hours
Ash2l-b-72 hours
Eif4a2PTC-48 hours
Eif4a2PTC-72 hours

B

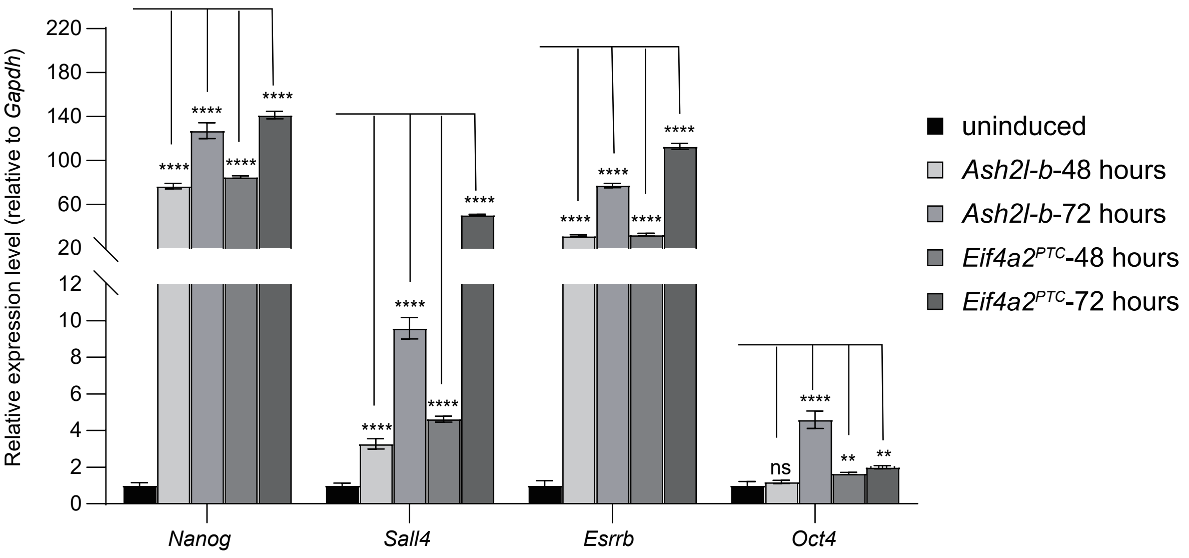

Relative expression level (relative to Gapdh)
220
180
140
100
60
20
12
10
8
6
4
2
0
ns
Nanog
Sall4
Esrrb
Oct4
uninduced
Ash2l-b-48 hours
Ash2l-b-72 hours
Eif4a2PTC-48 hours
Eif4a2PTC-72 hours

Figure S5

A. *Sox2*-KD

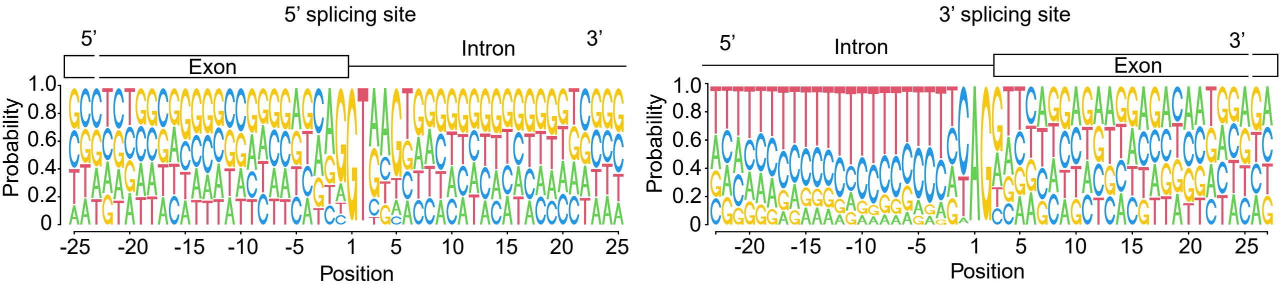

B. *hnRNPK*-KD

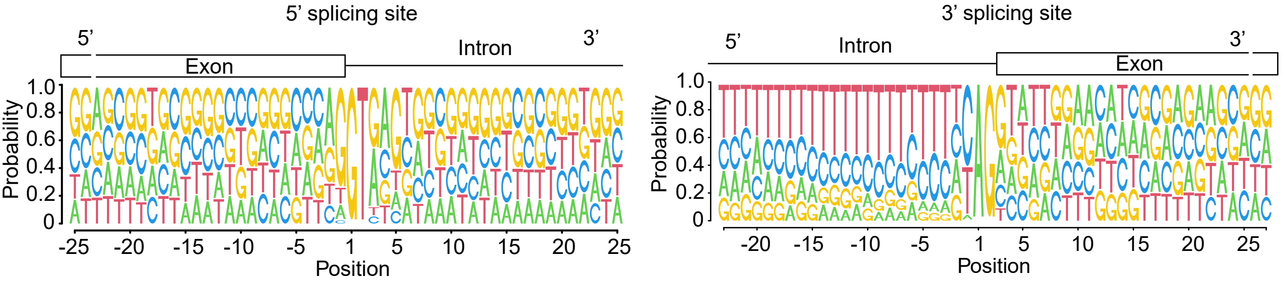

C. *Cdk9*-KD

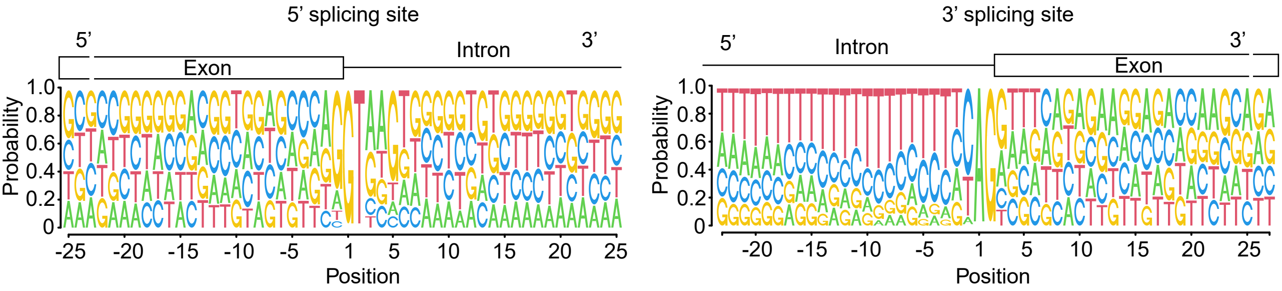

Supplement: Supplementary file 2 — Additional file 2. [file 13578_2024_1284_MOESM2_ESM.pdf]
